# Mol-CADiff: text-conditional molecule generation via causality-aware autoregressive diffusion

**DOI:** 10.1038/s41467-026-75702-5

**Published:** 2026-07-29

**Authors:** Md Atik Ahamed, Qiang Ye, Qiang Cheng

**Affiliations:** 1 Department of Computer Science, University of Kentucky, Lexington, Kentucky USA; 2 Department of Mathematics, University of Kentucky, Lexington, Kentucky USA; 3 Institute for Biomedical Informatics, University of Kentucky, Lexington, Kentucky USA

**Keywords:** Drug discovery, Pharmaceutics

## Abstract

The design of molecules with desired properties is a key challenge in drug discovery and materials science. Traditional methods rely on trial-and-error, while recent deep-learning approaches accelerate molecular generation. However, existing models struggle with generating molecules based on specific textual descriptions. We introduce Mol-CADiff, a diffusion-based framework that uses causal attention mechanisms for text-conditional molecular generation. Our approach explicitly models the causal relationship between textual prompts and molecular structures, overcoming limitations in existing methods. We enhance dependency modeling both within and across modalities, enabling precise control over the generation process. While primarily designed for text-guided tasks, this architecture inherently supports unconditional generation, providing the added capability to autonomously sample the broader chemical space without explicit constraints. Here we show that Mol-CADiff outperforms alternative methods in generating diverse, chemically valid molecules, with better alignment to specified properties, enabling more intuitive language-driven molecular design. By bridging these modalities, our framework provides a versatile method for drug discovery.

## Abstract (web summary)

Computational approaches to molecular design often explore only limited regions of the vast chemical space. This study presents a causality-aware diffusion model that generates valid and diverse molecules with or without text prompts, improving controllability in molecular design.

## Introduction

The design and discovery of novel molecules with desired properties is a foundational challenge in drug discovery, materials science, and chemical engineering. Traditional approaches to molecular design often rely on trial-and-error experimentation or computational methods that explore only limited regions of the vast chemical space. Recent advances in deep learning have shown promising results in automating and accelerating this process, enabling the generation of molecules with specified characteristics. However, molecular generation presents challenges that distinguish it from other generative tasks. Molecules must adhere to physical and chemical constraints to be valid and synthesizable. Moreover, the discrete nature of molecular structures, represented as graphs with atoms as nodes and bonds as edges, adds complexity compared to continuous domains like images or audio. Effective molecular generation requires models that can capture both local atomic interactions and global molecular properties while maintaining chemical Validity.

Early approaches to molecular generation relied on string-based representations such as SMILES^1^ and SELFIES^2^, which encode molecular structures as linear strings. While these representations are compact, they often struggle with maintaining chemical Validity in generation tasks^3,4^. More recent work has explored graph-based approaches that explicitly model molecular structures as graphs^5^. These methods have demonstrated significant advantages in generating valid, diverse, and novel molecular structures. Early graph-based approaches include variational autoencoders (VAEs)^6–8^, autoregressive (AR) models^9–12^, and flow-based models^13–15^. These methods have progressively improved in generating molecules with desired properties and structural constraints. A parallel line of research has focused on fragment-based approaches^16,17^, which decompose molecules into substructures and then recombine them, aligning with the fragment-based drug design paradigm in medicinal chemistry^18^. Recent work has also extended these approaches to 3D molecular generation by learning on topological surfaces and geometric structures^19^. More recently, graph generators have emerged utilizing advanced generative paradigms, such as discrete denoising diffusion in DiGress^20^ and diffusion mixtures in GruM^21^, to better capture complex structural distributions.

Recent advances in diffusion models^22,23^ have spurred their application to molecular generation^20,24^. Diffusion models have shown capabilities in generating high-quality samples across various domains^25^. In the molecular domain, these models offer a balance between exploration and Validity, addressing limitations of previous approaches. Developments include guided diffusion approaches for inverse molecular design^26^, structure-based drug design with equivariant diffusion models^27^, and object-aware equivariant models for transition state generation^28^. High-throughput property-driven generative design has also been demonstrated using diffusion-based approaches^29^.

Simultaneously, the success of large language models (LLMs) in natural language processing has inspired their application to molecular design^30–32^. Models like MolT5^30^ and ChemT5^33^ adapt the T5 architecture^34^ for molecular tasks, treating molecules as a specialized form of language. Mol-Instructions^35^ further enhances these capabilities by incorporating instruction-following abilities, enabling more directed molecular generation. More recently, approaches have expanded to leverage general-purpose LLMs, such as MolReGPT^36^, which utilizes ChatGPT for molecule-caption translation, and unified frameworks like Atomas^37^, which employ hierarchical adaptive alignment to better synchronize molecular and textual modalities. Recent work has also shown the potential of harnessing large language models for data-scarce learning scenarios, including polymer property prediction^38^.

Most recently, multimodal approaches that bridge textual descriptions and molecular structures have emerged^39–42^. These models aim to leverage the complementary strengths of language and molecular representations, enabling more intuitive and flexible control over generation through natural language instructions. 3M-Diffusion^43^ represents a significant advance in this direction by employing a latent diffusion approach that bridges molecular and textual modalities. However, existing diffusion-based approaches for controlled molecular generation often rely on simple architectures that fail to fully capture the complex dependencies between textual descriptions and molecular structures.

To better understand these dependencies, we conducted a per-molecule latent space causality analysis on the latent representations of the ChEBI-20 dataset. Each molecule’s latent vector was partitioned into contiguous groups to mimic the sequential tokenization strategy of our causal attention mask, enabling Granger causality tests for predictive dependencies.

**Fig. 1:**
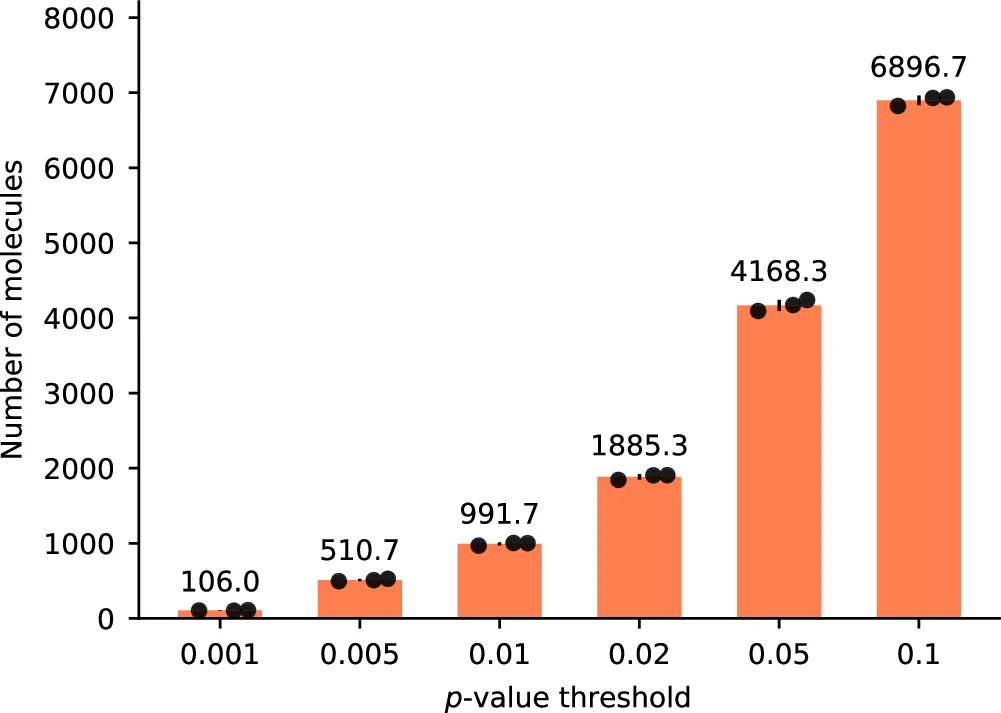
Number of ChEBI-20 molecules with significant Granger causal links. The bar plot displays the count of molecules identified with significant links in the latent space across varying *p*-value thresholds. Data are presented as mean ± s.d. over *n* = 3 independent runs with random partitioning. Individual data points are overlaid as dots. Orange bars represent mean values, black error bars represent s.d., and black dots represent individual runs. Significance was assessed using the sum-of-squared-residuals (SSR)-based Granger causality *F*-test. Nominal *p*-values were not adjusted for multiple comparisons. Source data are provided as a Source Data file.

As shown in Fig. 1, even under a stringent threshold (*p* < 0.001), a notable subset of molecules exhibits strong latent causal interactions. As the threshold relaxes (*p* < 0.1), nearly half of the molecules show at least one significant causal connection. These findings indicate that our latent spaces encode structured molecular causality, capturing predictive dependencies that support our attention mask design. Moreover, the tokenized text descriptions of molecules also have sequential dependencies.

In this work, we introduce Mol-CADiff, a causal attention diffusion framework for text-conditional molecular generation. Our approach addresses limitations of existing methods through several technical innovations. Specifically, we develop an attention-based denoising module for the diffusion network, enhancing dependency modeling both across modalities and within each modality. This contrasts with models like 3M-Diffusion^43^, which rely on MLP-based denoising. Furthermore, during training, we integrate partial tokens from the clean graph latent representation alongside conditional and noisy latent inputs, enriching contextual information for more effective molecule generation. Our approach explicitly models the causal relationship between text prompts and molecular graphs, ensuring structured generation. Finally, our comprehensive evaluation on standard molecular generation benchmarks demonstrates that Mol-CADiff outperforms existing methods across multiple metrics, including Validity, Novelty (the generation of molecules distinct from the reference structures), Uniqueness (the proportion of distinct molecular structures among the generated outputs), and alignment with specified properties. We show that this approach enables fine-grained control over molecular generation through natural language instructions for intuitive and accessible molecular design by domain experts without extensive programming knowledge.

## Results

### Datasets

We utilize four molecular datasets: PubChem^44^, ChEBI-20^39^, PCDes^40^, and MoMu^41^. To ensure a fair comparison, we follow prior studies on molecular structure generation^43,45–47^ and restrict the dataset to molecules with fewer than 30 atoms, aligning with the approach in^43^. Supplementary Table 1 presents the dataset statistics. PCDes and MoMu utilize the same training and validation set while maintaining different test sets. We present and compare the obtained results on the test set. In line with prior molecule-generation benchmarks, we restrict our dataset to small molecules containing fewer than thirty atoms. This aligns with common practice in generative modeling. Widely used datasets for small molecule generative models contain only relatively small molecules: for example, the QM9 dataset includes molecules with at most 9 heavy atoms^48^, while ZINC-based benchmarks such as ZINC-250K subset and MOSES filter for compounds up to ≈30 atoms^45,49^. Similarly, the GuacaMol benchmark suite focuses on generating lead-like (drug-like) compounds in this size range^50^.

In addition to prior studies, small molecules are prized in drug discovery for their favorable pharmacokinetic and biological properties. In particular, their low molecular weight and compact size enable them to readily penetrate cell membranes and reach intracellular targets^51^. The vast majority of medicines are small molecules, underscoring their real-world relevance. Over 90% of marketed drugs are small-molecule compounds^52^, and in the past decade, roughly two-thirds of new FDA drug approvals have been small-molecule therapeutics^53^. This dominance reflects the broad therapeutic scope of small molecules. They have consistently delivered medical breakthroughs across many disease areas, from antibiotics and analgesics to anticancer therapies.

Therefore, confining our data to molecules below 30 atoms maintains chemical relevance and consistency with established small-molecule generation benchmarks.

### Metrics

We evaluate model performance on text-guided conditional and unconditional molecule generation. For text-guided generation, we follow prior works^30,33,35,43^ and assess (i) Similarity, the percentage of generated molecules matching the ground truth with MACCS^54^ cosine similarity with a threshold of 0.5; (ii) Novelty, the fraction of qualified molecules with $f(G,\hat{G}) < 0.8$, where *f* measures MACCS and a molecule is identified as qualified if $f(G,\hat{G})\ge 0.5$; (iii) Diversity, the average pairwise distance 1 − *f*(⋅,⋅) between qualified molecules; and (iv) Validity, the percentage of chemically valid molecules.

For unconditional generation, we employ the GuacaMol benchmarks^50^ and evaluate (i) Uniqueness, the ratio of distinct molecules; (ii) Novelty, the fraction of molecules not present in the training set; (iii) KL Divergence, which quantifies alignment with training set distributions; and (iv) Fréchet ChemNet Distance (FCD)^55^, which compares feature distributions in ChemNet. Since lower values indicate better performance for raw KL divergence and FCD, we apply the exponential transformation described in^50^ to map these metrics to normalized scores. Consequently, all metrics range from 0 to 1 and are reported as percentages, where higher values indicate superior performance. However, as observed in Tables 1, 2, models often exhibit trade-offs, excelling in specific metrics (e.g., MolT5-large in Similarity) while underperforming in others (e.g., lower Novelty and Diversity). To provide an overall comparison, we introduce an (v) Overall metric, defined as the average of all individual metrics. This offers a balanced assessment of a model’s general performance rather than over-emphasizing a single dimension.

**Table 1:** Quantitative performance on conditional molecule generation

	ChEBI-20					PubChem					PCDes					MoMu				
Methods	Sim.	Nov.	Div.	Val.	Ove.	Sim.	Nov.	Div.	Val.	Ove.	Sim.	Nov.	Div.	Val.	Ove.	Sim.	Nov.	Div.	Val.	Ove.
MolT5-small	73.32	31.43	17.22	78.27	50.06	68.36	20.63	9.32	78.86	44.29	64.84	24.91	9.67	73.96	43.35	16.64	97.49	29.95	60.19	51.07
MolT5-base	80.75	32.83	17.66	84.63	53.97	73.85	21.86	9.89	79.88	46.37	71.71	25.85	10.50	81.92	47.50	19.76	97.78	29.98	68.84	54.09
MolT5-large	96.88	12.92	11.20	98.06	54.77	91.57*	20.85	9.84	95.18	54.36	88.37*	20.15	9.49	96.48	53.62	25.07*	97.47	30.33	90.40	60.82
Atomas	96.98*	41.52	26.98	98.03	65.88	90.33	44.39	30.11	94.88	64.93	85.88	34.44	20.12	97.89	59.58	23.05	97.88	34.01	89.88	61.21
ChemT5-small	96.22	13.94	13.50	96.74	55.10	89.32	20.89	13.10	93.47	54.19	86.27	23.28	13.17	93.73	54.11	23.25	96.97	30.04	88.45	59.68
ChemT5-base	95.48	15.12	13.91	97.15	55.42	89.42	22.40	13.98	92.43	54.56	85.01	25.55	14.08	92.93	54.39	23.40	97.65	30.07	87.61	59.68
Mol-Instruction	65.75	32.01	26.50	77.91	50.54	23.40	37.37	27.97	71.10	39.96	60.86	35.60	24.57	79.19	50.06	14.89	97.52	30.17	68.32	52.73
3M-Diffusion	87.09	55.36	34.03	100.0*	69.12	87.05	64.41	33.44	100.0*	71.22	81.57	63.66	32.39	100.0*	69.41	24.62	98.16	37.65	100.0*	65.11
Mol-CADiff	81.92	67.35*	75.69*	100.0*	81.24*	78.35	71.97*	75.39*	100.0*	81.43*	71.41	74.77*	75.45*	100.0*	80.41*	24.41	98.34*	83.30*	100.0*	76.51*

Comparative analysis across four benchmark datasets. Metrics include Similarity (Sim.), Novelty (Nov.), Diversity (Div.), and Validity (Val.), with Overall (Ove.) representing their arithmetic mean. All results are percentages (higher is better). Mol-CADiff outperforms methods in Novelty, Diversity, and Validity while maintaining competitive Similarity, achieving the highest Overall scores across all datasets. An asterisk (*) indicates the best performance in each column.

**Table 2:** Quantitative performance on unconditional generation

	ChEBI-20						PubChem					
Methods	Uni.	Nov.	KL	FCD	Val.	Ove.	Uni.	Nov.	KL	FCD	Val.	Ove.
CharRNN	72.46	11.57	95.21	75.95	98.21	70.68	63.28	23.47	90.72	76.02*	94.09	69.52
VAE	57.57	47.88	95.47	74.19	63.84	67.79	44.45	42.47	91.67	55.56	94.10	65.65
AAE	1.23	1.23	38.47	0.06	1.35	8.47	2.94	3.21	39.33	0.08	1.97	9.51
LatentGAN	66.93	57.52	94.38	76.65	73.02	73.70	52.00	50.36	91.38	57.38	53.62	60.95
BwR	22.09	21.97	50.59	0.26	22.66	23.51	82.35	82.34	45.53	0.11	87.73	59.61
HierVAE	82.17	72.83	93.39	64.32	100.0*	82.54	75.33	72.44	89.05	50.04	100.0*	77.37
PS-VAE	76.09	74.55	83.16	32.44	100.0*	73.25	66.97	66.52	83.41	14.41	100.0*	66.26
GruM	83.10	75.20	94.85	74.15	99.40	85.34	84.90	79.20	94.60	67.85	99.50	85.21
DiGress	82.40	74.50	94.10	72.60	98.70	84.46	83.85	78.95	93.90	66.95	98.90	84.51
3M-Diffusion	83.04	70.80	96.29*	77.83*	100.0*	85.59	85.42	81.20	92.67	58.27	100.0*	83.51
Mol-CADiff	85.90*	79.11*	94.69	74.45	100.0*	86.83*	87.79*	83.23*	95.19*	71.65	100.0*	87.57*

Comparison across ChEBI-20 and PubChem datasets. Metrics include Uniqueness (Uni.), Novelty (Nov.), transformed KL Divergence (KL), transformed Fréchet ChemNet Distance (FCD), and Validity (Val.). Ove. denotes the arithmetic mean. All results are percentages (higher is better). Mol-CADiff achieves the highest Overall performance, demonstrating a strong balance between Validity and distributional alignment. An asterisk (*) indicates the best performance in each column.

### Implementation details

Here, we outline our implementation for conditional and unconditional molecule generation and briefly introduce the baseline models for comparison.

#### Baselines

For conditional/instruction-guided molecule generation, we evaluate Mol-CADiff against multiple baseline models: MolT5^30^, ChemT5^33^, Mol-Instruction^35^, 3M-Diffusion^43^, Atomas^37^, and MolReGPT^36^ (on one dataset), including variations of MolT5 (small, base, and large) and ChemT5 (small and base).

For unconditional molecule generation, we compare Mol-CADiff against a broad range of generative methods, including Variational Autoencoder-based models such as VAE^56^, AAE^57^, BwR^58^, HierVAE^16^, and PS-VAE^17^, as well as CharRNN^59^, LatentGAN^60^, and 3M-Diffusion^43^. We also evaluate performance against recent graph generators, specifically DiGress^20^ and GruM^21^. Our evaluation follows the experimental protocols established by 3M-Diffusion, including the training, validation, and test set splits. To ensure a fair comparison and maintain protocol consistency, we report baseline results directly from the original publications, thereby avoiding potential discrepancies or confounding factors arising from reimplementation or hyperparameter mismatch.

#### Experimental settings

For the graph encoder, we employ GIN^61^ to obtain latent representations of molecular graphs, while Sci-BERT^62^ is used for compressed text representation, and HierVAE^16^ is used as the graph decoder. In the CaDiff module, we integrate graph and text information autoregressively, ensuring proper causal dependencies. For both conditional and unconditional generation tasks, we set the diffusion timesteps to *T* = 100 during training, with *S*_T_ = 50 for sampling in conditional generation and 25 for unconditional generation on the ChEBI-20 dataset. The model is optimized using the Adam optimizer^63^ with an initial learning rate of 0.001, training for 1000 epochs.

To incorporate classifier-free guidance^64^, we randomly drop the conditional embeddings with a probability of 0.1 during training and inference for conditional generation, while for unconditional generation, this probability is set to 1.0, effectively removing all contextual information. Additional settings, including the number of attention blocks and the proportion of clean tokens, are analyzed in the ablation study. Our implementation is based on PyTorch^65^, and all experiments were conducted on NVIDIA A100 GPUs.

### Quantitative comparison

For quantitative evaluation, we report the results of conditional generation in Table 1. Our method consistently outperforms all baselines in Overall performance by a large margin. In addition to excelling in Overall metrics, Mol-CADiff maintains strong Similarity across all four datasets. It achieves substantial improvements in Novelty and Diversity, demonstrating its effectiveness in generating chemically valid, novel, and diverse molecules while preserving Similarity to the given condition.

For unconditional generation, following 3M-Diffusion, we present results on the ChEBI-20 and PubChem datasets in Table 2. Here too, Mol-CADiff achieves the highest Overall performance, surpassing baseline methods. Furthermore, it exhibits strong Uniqueness, Novelty, KL Divergence, and FCD, either outperforming baselines or maintaining competitive results. These results reinforce Mol-CADiff’s superiority in both conditional and unconditional molecular generation, demonstrating its robustness, efficiency, and ability to generate effective molecular structures.

While Mol-CADiff may not always achieve the highest raw Similarity scores compared to some baselines, it consistently excels in generating novel, diverse, and valid molecules across all datasets. This strength is crucial, as generative molecular design prioritizes the creation of chemically valid, novel compounds that explore uncharted regions of chemical space, rather than merely replicating known structures^66,67^. In other words, the goal is to go beyond the training distribution and discover compounds that meet design criteria while venturing into unexplored chemical territory, an essential objective in drug discovery. Models that prioritize Similarity alone risk overfitting the training data, resulting in reduced Diversity and Novelty among generated molecules^68^. This overemphasis can lead to mode collapse, where the model repeatedly generates a narrow range of highly similar structures instead of exploring the broader chemical space. In contrast, Mol-CADiff strikes an effective balance between conditional fidelity (adherence to design constraints) and structural innovation, demonstrating a strong ability to generalize beyond seen molecules and uncover meaningful new candidates.

This balance is particularly valuable in drug discovery and materials science, where identifying rare or previously unobserved compounds often has more impact than rediscovering known ones^69^. For example, even if a generated molecule is chemically valid, relying on known core scaffolds may reduce patentability or render it less commercially attractive^69^. By explicitly incorporating causal dependencies via our attention-mask design, Mol-CADiff organizes its latent space in a way that supports both high-fidelity conditioning and molecular innovation.

### Qualitative comparison

**Fig. 2:**
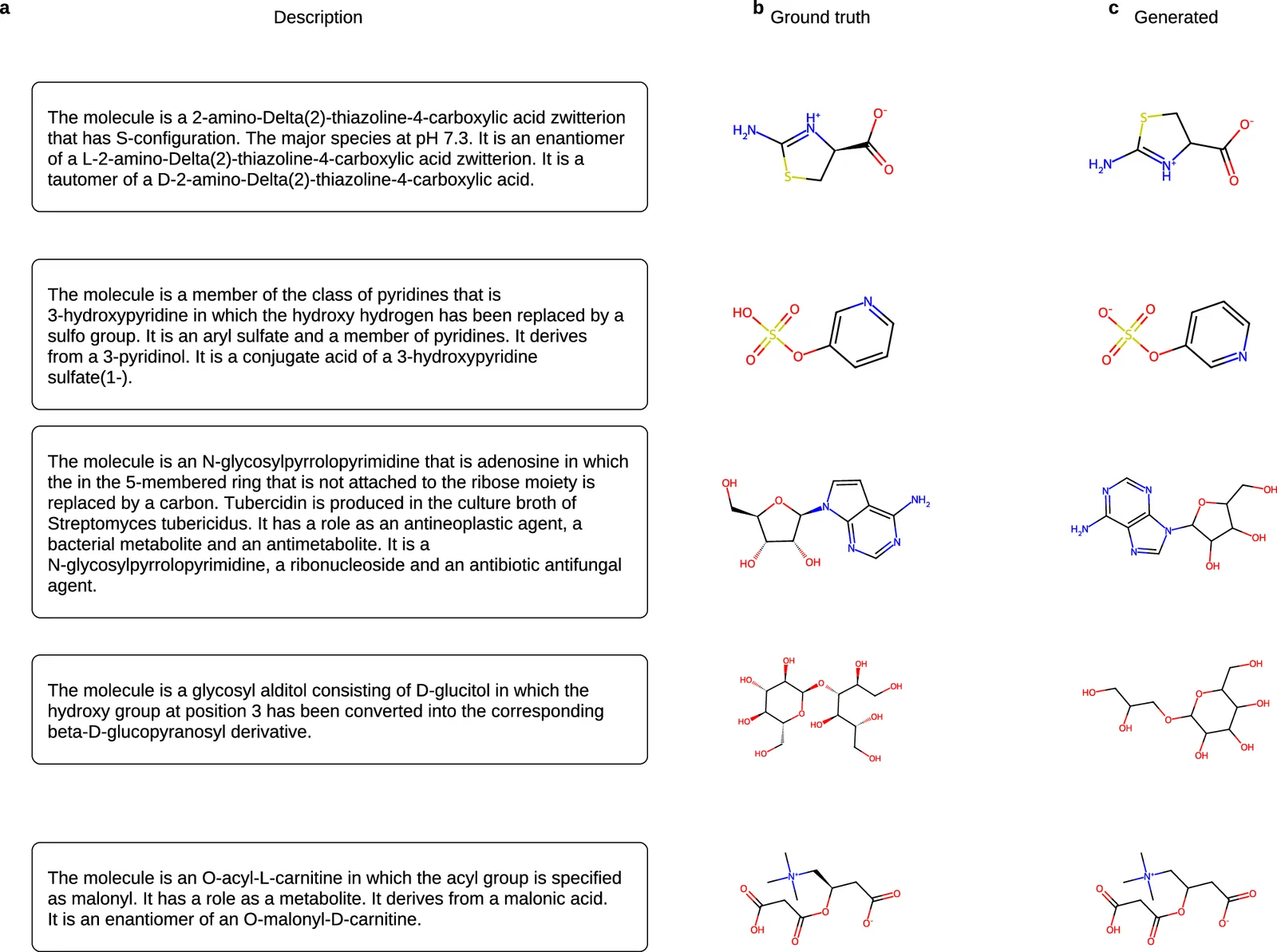
Qualitative evaluation of text-to-molecule generation. Representative examples from the held-out ChEBI-20 test set are shown alongside their corresponding input text prompts. **a** Input text prompts shown in white boxes with black borders. **b** Ground truth molecules corresponding to the input prompts. **c** Generated molecules, demonstrating accurate alignment with the textual descriptions and high structural fidelity compared with the Ground truth molecules. Atom and bond colors follow the molecular drawing convention used in the figure.

**Fig. 3:**
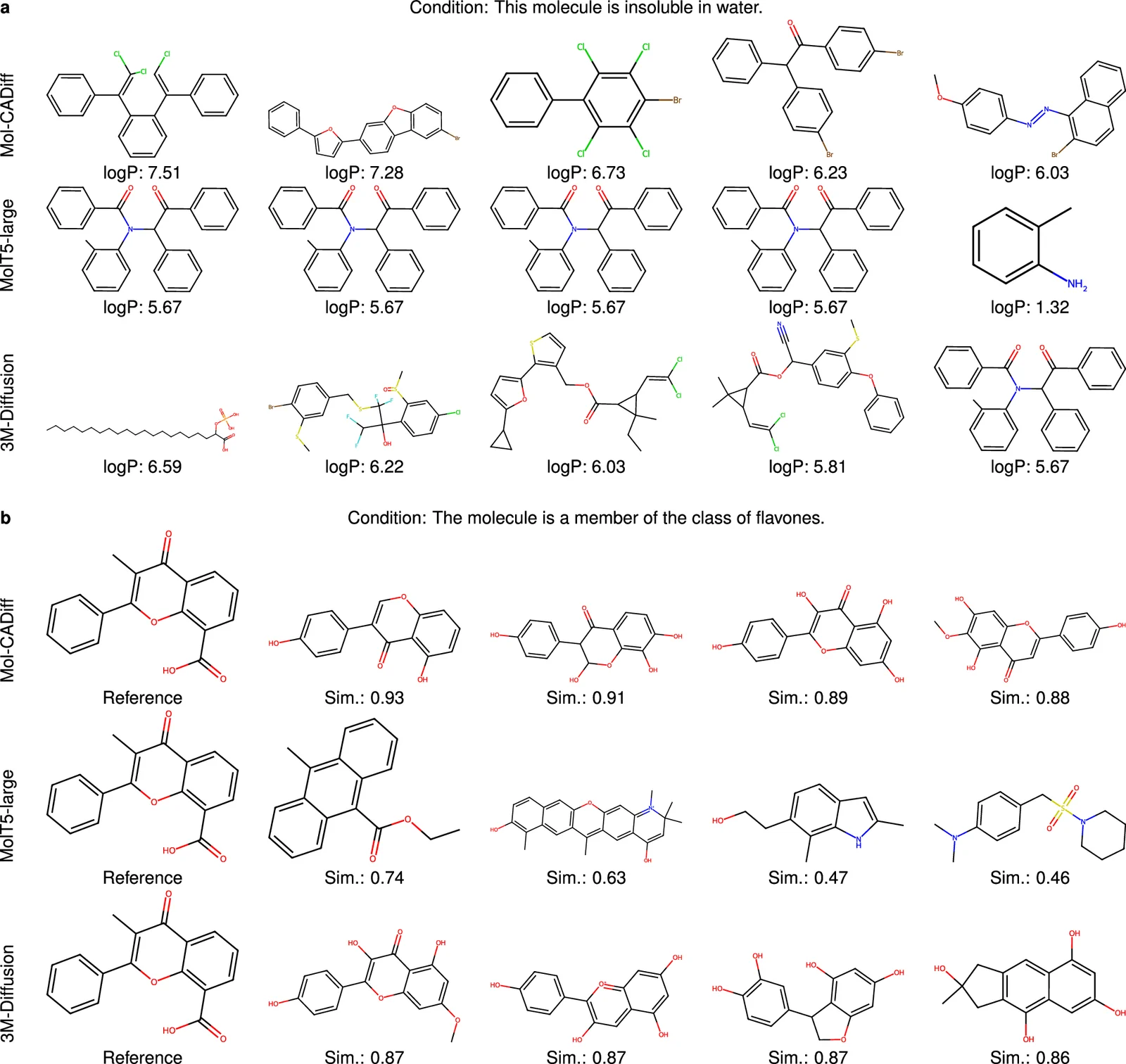
Case studies of property-directed molecular generation for insolubility and flavone scaffolds. Representative top-5 molecules generated by Mol-CADiff, 3M-Diffusion, and MolT5-large are shown for the prompts “This molecule is insoluble in water,” and “The molecule is a member of the class of flavones.” **a** Molecules generated under the insolubility prompt are displayed with their corresponding logP scores. **b** Molecules generated under the flavone scaffold are displayed with the reference structure and Similarity (Sim.) scores. Generations are targeted toward high logP (hydrophobicity) and structural Similarity to reference flavones. These examples illustrate the models' ability to satisfy specific chemical constraints while maintaining structural Validity. Atom and bond colors follow the molecular drawing convention. No statistical hypothesis testing was performed for this figure.

We present qualitative results demonstrating the efficacy of our method in Fig. 2, where we visualize five randomly sampled molecules generated by Mol-CADiff alongside their corresponding ground truth structures. It is evident that the generated molecules closely resemble the ground truth, faithfully capturing the textual descriptions provided. This visual similarity underscores our method’s ability to effectively leverage textual conditions that reflect the desired properties. Furthermore, we conduct detailed case studies in Fig. 3, comparing Mol-CADiff with two best-performing methods, namely 3M-Diffusion and MolT5-large, under specific textual instructions. As illustrated, Mol-CADiff consistently outperforms both baselines by generating molecules that better satisfy the given custom conditions.

For instance, when instructed to generate molecules insoluble in water, Mol-CADiff produces molecules with higher logP values, clearly surpassing the baselines in property-steering accuracy. MolT5-large frequently generates near-identical molecules across multiple sampling attempts; this lack of variance is consistent with its lower Diversity scores reported in Table 1 and corroborates observations from prior work, such as 3M-Diffusion^43^. Similarly, when generating molecules belonging to the flavone class, our method achieves higher structural Similarity to the reference molecules compared to competing methods. These qualitative analyses further validate Mol-CADiff’s superiority in generating high-quality, conditionally accurate molecules.

Additional results, including more generated molecules and extended case studies, are provided in the Supplementary Figs. 2–5.

### Pharmaceutically relevant property distributions

**Fig. 4:**
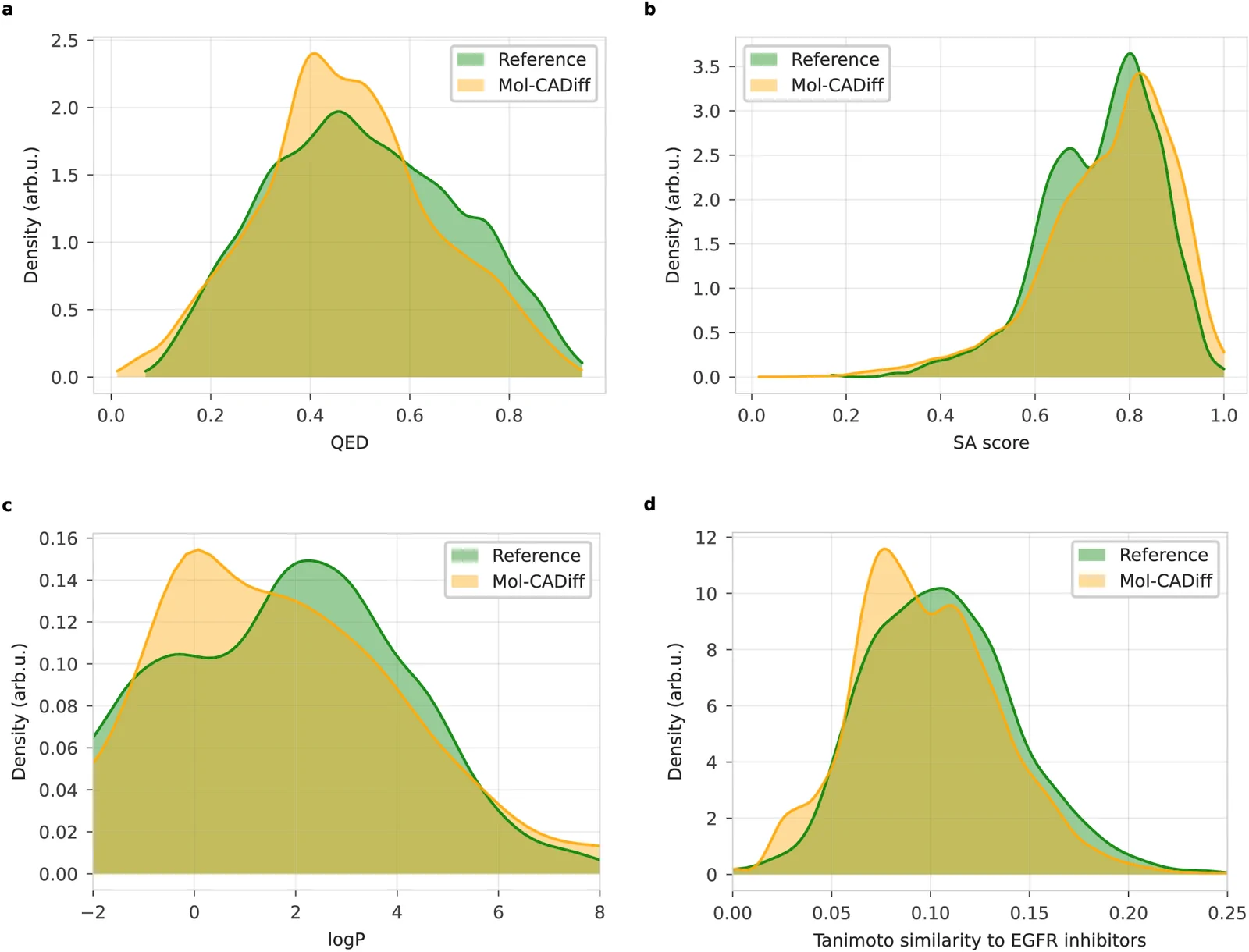
Distributions of pharmaceutically relevant properties and target structural similarity. **a–c** Probability density estimates of molecular property values for molecules generated by Mol-CADiff versus reference molecules for **a** QED, **b** SA score, and **c** logP. Greater distribution overlap indicates closer alignment with the reference chemical space. For panel (**c**), no statistical hypothesis testing was performed. **d** Comparison of Tanimoto similarity to EGFR inhibitors using ECFP4 between reference molecules and those generated by Mol-CADiff, evaluated against a set of known EGFR inhibitors. The high degree of overlap indicates that the model preserves the target-relevant structural profiles. Green indicates the reference distribution, and orange indicates the Mol-CADiff distribution. Density values are shown in arbitrary units. Source data are provided as a Source Data file.

To further validate the practical utility of the molecules generated by Mol-CADiff, we examine the distributions of three widely used pharmaceutically relevant properties: quantitative estimate of drug-likeness (QED), synthetic accessibility (SA), rescaled to [0, 1] where 1 represents maximum accessibility, and lipophilicity (logP). For each generated molecule and its reference counterpart, we computed these properties using RDKit and compared their empirical distributions. Figure 4 shows kernel density estimates of the distributions. In each plot, the x-axis represents the property value, while the y-axis represents the estimated probability density. The shaded regions correspond to the probability mass. Greater overlap between the shaded curves indicates closer alignment with the reference distribution.

The results demonstrate that our method closely matches the reference distributions across all three properties. For QED (Fig. 4a), our model produces distributions that are well aligned with the reference, ensuring drug-like relevance. For SA (Fig. 4b), the overlap is nearly perfect, highlighting the model’s ability to generate molecules that are synthetically feasible. Similarly, for logP (Fig. 4c), the generated molecules follow the overall shape of the reference distribution, capturing the lipophilicity profile critical for bioavailability. Overall, these results confirm that beyond achieving high Novelty and Diversity, Mol-CADiff generates molecules whose pharmaceutical property distributions remain consistent with real-world compounds, strengthening the case for its practical applicability in drug discovery.

### Ligand-based target-relevant validation

To complement the evaluation of chemical Validity and drug-like properties, we conducted a ligand-based validation to assess whether Mol-CADiff generates molecules with target-relevant potential. As a representative case study, we considered the epidermal growth factor receptor (EGFR), a well-studied oncology target with multiple approved inhibitors. We computed Extended-Connectivity Fingerprint, diameter 4 (ECFP4) similarities, which are Tanimoto similarities calculated using ECFP4 fingerprints, to a set of known EGFR inhibitors for both: (i) ground-truth molecules from the ChEBI-20 dataset and (ii) molecules generated by Mol-CADiff. This was done to evaluate whether the model preserves target-specific motifs.

Figure 4d shows that the similarity distributions of generated and ground-truth molecules to EGFR inhibitors almost completely overlap, indicating that our model reproduces the same target-relevant chemical patterns present in the reference dataset. This further confirms that Mol-CADiff is not generating molecules arbitrarily but instead captures pharmacologically meaningful regions of chemical space.

### Ablation study

We also analyze the impact of key components in our method through ablation studies. To isolate the effect of each parameter or component, we vary one factor at a time while keeping all others fixed. We conduct our ablation study mostly on the PubChem dataset, as it offers a balanced trade-off between dataset size and computational efficiency. We explicitly indicate instances where alternative datasets are employed.

### AR step decay

**Fig. 5:**
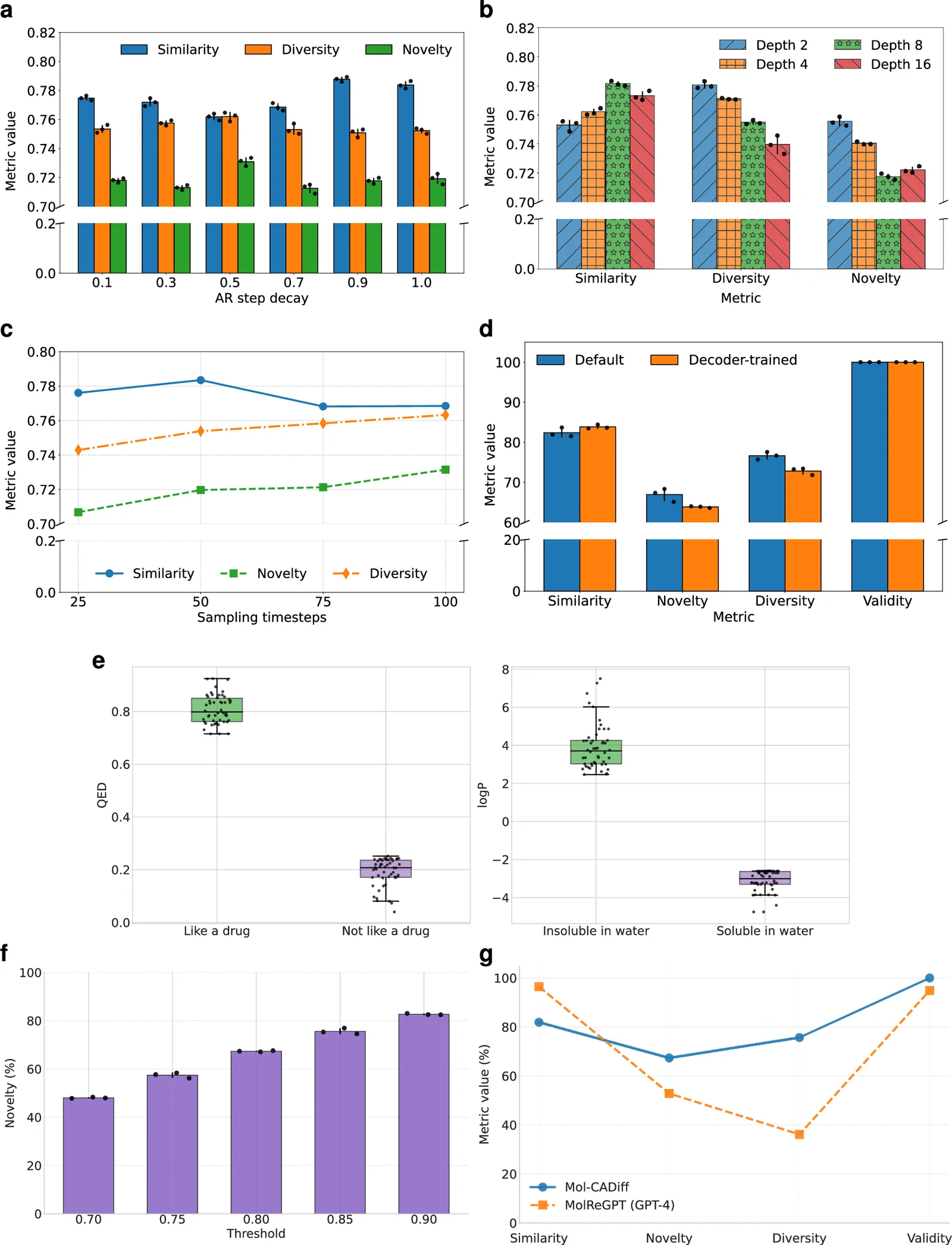
Ablation studies and sensitivity analysis of model hyperparameters and property interventions. **a** Impact of the AR decay factor on model metrics. **b** Effect of the number of attention blocks. **c** Sensitivity to sampling steps, *S*_T_. **d** Comparison of the default Mol-CADiff model against joint training of the decoder and diffusion model. **e** Intervention-based boxplots demonstrating text-driven shifts in QED and logP. Prompts for drug-likeness shift the distribution toward higher QED, while prompts for insolubility increase logP. For boxplots, the center line represents the median, the box limits represent the upper and lower quartiles, and the whiskers represent 1.5 × the interquartile range. For panel (**e**), each boxplot summarizes *n* = 50 valid generated molecules per prompt condition; each point represents one generated molecule, a computational output rather than a biological or technical replicate. For panel (**e**), no statistical hypothesis testing was performed. **f** Percentage of novel molecules generated across varying thresholds. **g** Performance comparison of Mol-CADiff with the GPT-4-based baseline MolReGPT. Colors correspond to the metrics, model variants, property settings, novelty thresholds, or comparison methods indicated in the corresponding panel legends. In panels (**a**, **b**, **d**, **f**), bar heights represent the mean and error bars indicate mean ± s.d. from *n* = 3 independent runs. Panels (**c**, **g**) show results from a single representative run. Source data are provided as a Source Data file.

As discussed in Subsection Autoregressive steps formulation, the AR step sizes depend on the decay factor *γ*. Figure 5a shows its impact on Similarity, Diversity, and Novelty in the PubChem dataset. Similarity remains relatively stable but improves slightly for higher values of *γ*, suggesting that larger AR steps help maintain structural coherence. Diversity peaks around *γ* = 0.5 but declines at large values, indicating that a balanced step size prevents excessive fragmentation or oversimplification. Novelty also peaks at *γ* = 0.5, showing that moderate decay encourages the exploration of new molecular structures.

### Attention blocks

We analyze the impact of attention blocks (2, 4, 8, 16) on Similarity, Diversity, and Novelty in the PubChem dataset, excluding Validity (always 100.0%). See Fig. 5b. Similarity improves with more attention blocks, peaking at 8, suggesting that deeper architectures enhance molecular coherence. However, Diversity and Novelty decline beyond 2 blocks, indicating that excessive depth may reduce structural variation. To balance performance and efficiency, we set 8 blocks as the default, as increasing depth further raises the parameter count and computational cost.

### Sampling time steps

We analyze the impact of different sampling timesteps (*S*_T_) during inference on the quality of generated molecules. Specifically, we evaluate Similarity, Novelty, and Diversity across: *S*_T_ ∈ {25, 50, 75,100}. As illustrated in Fig. 5c, Similarity is maximized at *S*_T_ = 50, suggesting that an adequate number of sampling steps improves the structural accuracy of generated molecules. Novelty and Diversity exhibit an increasing trend as *S*_T_ increases, with the highest values observed at *S*_T_ = 100. This indicates that a greater number of sampling steps introduces more novel and diverse molecules. However, beyond *S*_T_ = 50, Similarity slightly decreases, likely due to excessive noise reduction leading to potential loss of structural integrity. Based on these observations, we set: *S*_T_ = 50 as the default configuration for conditional generation, as it balances Similarity, Novelty, and Diversity while maintaining computational efficiency.

### Effect of joint decoder training during diffusion learning

To evaluate whether jointly training the graph decoder during the diffusion learning stage improves the alignment between generated latents and the decoder’s latent space, we conduct an additional ablation study on the ChEBI-20 dataset. In this variant, the graph decoder is updated simultaneously with the diffusion model, rather than remaining fixed after the initial VAE pretraining stage.

Figure 5d presents the comparative results. Joint decoder training yields equivalent chemical Validity (100%) and only marginal fluctuations in Similarity, Novelty, and Diversity. These minor differences do not indicate a systematic improvement in performance. This demonstrates that the diffusion denoiser effectively learns to produce latents well-aligned with the pretrained encoder manifold, and that the VAE decoder is robust to minor variations in the latent distribution. Given the lack of significant benefits and the increased computational overhead, we retain the fixed pretrained decoder as our default configuration.

### Intervention-based ablation

To examine whether text conditions meaningfully influence the molecular properties produced by Mol-CADiff, we perform a controlled intervention-based ablation using contrasting prompts. For drug-likeness, we compare the top 50 generated molecules conditioned on “The molecule is like a drug” versus “The molecule is not like a drug.” We quantify the results using the QED score, where higher values indicate structures with greater drug-like relevance. As shown in Fig. 5e, conditioning on the positive drug-like prompt produces molecules with substantially higher QED scores, while the negative prompt shifts the distribution toward significantly lower values, confirming that the text effectively modulates the generative trajectory toward or away from drug-like chemical space.

Similarly, for solubility, we contrast the prompts “The molecule is insoluble in water” and “The molecule is soluble in water,” using logP as the evaluative metric (where higher logP indicates increased hydrophobicity). The “insoluble” prompt yields molecules with significantly higher logP, whereas the “soluble” prompt produces molecules with much lower logP values. Taken together, these intervention-based tests indicate that textual conditions exert a systematic and property-congruent influence on the generated structures. These observed trends, including higher QED for drug-like prompts and higher logP for insolubility prompts, align with established chemical principles. These results further validate the effectiveness of text-guided generation within the Mol-CADiff framework.

### Ablation on novelty threshold using MACCS similarity

We conduct an ablation study to assess the sensitivity of our Novelty metrics across MACCS-based Similarity cutoffs of 0.70, 0.75, 0.80, 0.85, and 0.90. Figure 5f reports the percentage of generated molecules classified as novel at each threshold. As expected, the Novelty rate increases monotonically as the cutoff becomes more relaxed (as implied by the Novelty definition). Specifically, the Novelty percentage rises from 48.04% at a cutoff of 0.70 to 82.67% at a cutoff of 0.90. At the standard 0.80 cutoff frequently adopted in prior literature, Mol-CADiff achieves 67.35% Novelty. This confirms that the model’s capacity for generating novel structures is significant and consistent under standard evaluation settings.

### Comparison with GPT-4-based MolReGPT

We additionally compare Mol-CADiff with another LLM-based approach, MolReGPT^36^, instantiated with GPT-4 (version 0314) on the ChEBI-20 dataset. We evaluate the same four conditional generation metrics used in our main analysis: Similarity, Novelty, Diversity, and Validity. The results are summarized in Fig. 5g. We observe that MolReGPT achieves Similarity scores comparable to MolT5-large and Atomas, reflecting a tendency to generate structures that remain very close to the ground-truth molecules. However, this high Similarity comes at the cost of substantially reduced Novelty and Diversity, indicating that GPT-4 primarily explores regions of chemical space already well-covered by the training data.

In contrast, Mol-CADiff attains higher Novelty and Diversity while maintaining perfect Validity and competitive Similarity. This trade-off aligns with our core design goal: Mol-CADiff is engineered to explore chemically plausible yet novel molecules rather than simply reproducing near-duplicates of the reference set. Since MolReGPT reports results exclusively on the ChEBI-20 dataset, we present our comparison on this benchmark to ensure a fair evaluation; the observed trends are consistent with the multi-dataset comparisons detailed in our main results.

### Effect of partial graph latent representation

**Fig. 6:**
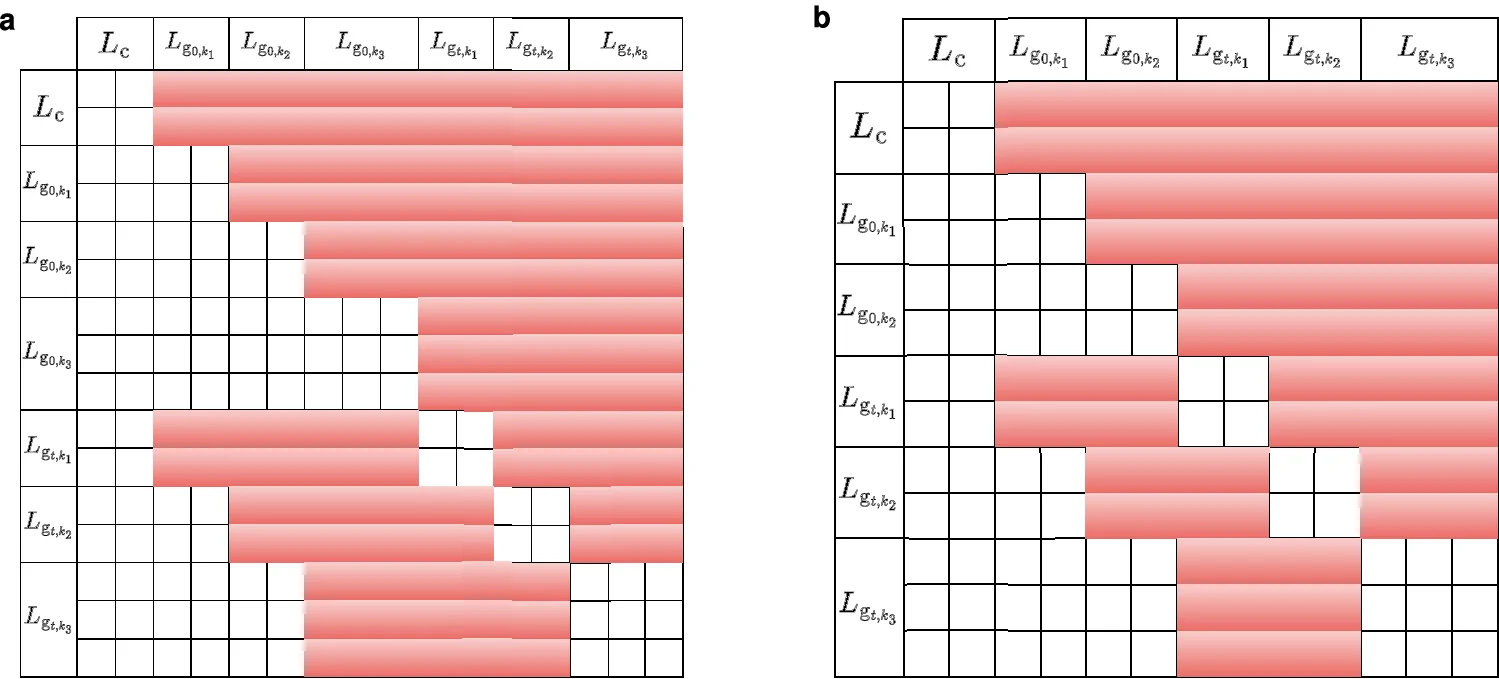
Attention masking strategies for full and partial graph latent representations. Visualization of mask construction for **a** full versus **b** partial graph latents ${L}_{{{{\rm{g}}}}_{0}}$. The masks illustrate a three-step AR process with token sizes of 2, 2, and 3. Setting **b** excludes ${L}_{{{{\rm{g}}}}_{0,{k}_{3}}}$ to reduce computational overhead while maintaining high generative performance. White cells indicate unmasked entries that are available for attention, red shaded cells indicate masked entries that are not attended to, and black grid lines separate latent-token positions.

We analyze the impact of including the full vs. partial graph latent representation ${L}_{{{{\rm{g}}}}_{0}}$. Figure 6 illustrates an example attention mask for three AR steps, with token sizes of 2, 2, and 3. The left side represents the full latent ${L}_{{{{\rm{g}}}}_{0}}$, which incorporates ${L}_{{{{\rm{g}}}}_{0,{k}_{3}}}$, while the right side depicts the partial setting, where only ${L}_{{{{\rm{g}}}}_{0,{k}_{1}}}$ and ${L}_{{{{\rm{g}}}}_{0,{k}_{2}}}$ are included. Table 3 presents the results across all datasets. On ChEBI-20 and PubChem, the partial setting yields better Overall performance compared to full inclusion. However, for MoMu, Similarity slightly improves under the full setting, while for PCDes, full inclusion achieves marginal gains in Novelty and Diversity. Despite these variations, partial inclusion of *L*_g_ offers computational efficiency by reducing token processing. Considering the trade-offs between performance and efficiency, we adopt the partial ${L}_{{{{\rm{g}}}}_{0}}$ setting.

**Table 3:** Ablation of graph latent representation (${L}_{{{{\rm{g}}}}_{0}}$) settings

	ChEBI-20					PubChem				
Setting	Sim.	Nov.	Div.	Val.	Ove.	Sim.	Nov.	Div.	Val.	Ove.
Partial	81.92	67.35	75.69	100.0	81.24*	78.35	71.97	75.39	100.0	81.43*
Full	82.56	64.81	71.75	100.0	79.78	76.35	73.60	75.65	100.0	81.40
	PCDes					MoMu				
Setting	Sim.	Nov.	Div.	Val.	Ove.	Sim.	Nov.	Div.	Val.	Ove.
Partial	71.41	74.77	75.45	100.0	80.41	24.41	98.34	83.30	100.0	76.51
Full	70.72	76.28	76.07	100.0	80.77*	25.56	98.02	82.98	100.0	76.64*

Comparison between Partial and Full inclusion of these tokens in the cross-attention mechanism across four datasets. Results are reported as percentages (higher is better). The Partial setting achieves competitive Overall (Ove.) performance with higher efficiency, and thus is used as our default configuration. An asterisk (*) indicates the best Overall performance in each column.

## Discussion

We present Mol-CADiff, a causality-aware autoregressive diffusion framework designed for generating high-quality molecular structures from textual descriptions. Demonstrating strong performance across four standard benchmarks (ChEBI-20, PubChem, PCDes, and MoMu), Mol-CADiff excels particularly in generating chemically valid, novel, and diverse molecules while maintaining high fidelity to input instructions. Mol-CADiff substantially enhances the generative process by explicitly modeling the causal dependencies between text prompts and molecular graphs. By bridging the gap between continuous diffusion and discrete sequence modeling, Mol-CADiff effectively produces molecules that are not only structurally valid but also aligned with complex property descriptions.

### Technical advantages and causal modeling:

Mol-CADiff stands out from current language-based and graph-based generation techniques by offering a superior balance between Similarity, Novelty, and Diversity, primarily due to its integration of causal attention within the denoising process. Unlike previous methods that treat molecular generation as either a simple translation task or an unstructured point-cloud generation, our approach integrates these paradigms via a causal mask that respects the sequential dependencies of molecular topology. Our latent space analysis reveals that molecular representations possess inherent causal structures; by leveraging these dependencies through our attention mechanism, we ensure that the denoising of current tokens is guided by chemically relevant context from previous steps. In previous methods, such as standard latent diffusion, the lack of explicit causal constraints often leads to the generation of valid but generic structures. As a result, the generated molecules might not be effectively tailored to the specific constraints of the text prompt. Furthermore, our framework learns a generative model that excels in exploring novel regions of chemical space, achieving significantly higher Diversity compared to Large Language Model baselines like MolReGPT.

### Interpretation of chemical semantics

Mol-CADiff excels in interpreting high-level chemical semantics, surpassing the capabilities of models that rely solely on statistical correlations. In the context of drug discovery, generating molecules that strictly adhere to property constraints (e.g., solubility, drug-likeness) is critical. Our intervention-based ablation studies demonstrate that Mol-CADiff is capable of directionally steering molecular generation based on specific prompts. For instance, instructing the model to generate “insoluble” molecules results in a systematic shift toward higher logP values, while prompts for “drug-likeness” yield molecules with significantly improved QED scores. This confirms that the text conditioning in Mol-CADiff exerts a meaningful, chemically consistent influence on the structure, rather than merely retrieving nearest neighbors from the training set. Additionally, the distribution of pharmaceutical properties, including synthetic accessibility and lipophilicity, in our generated samples closely mirrors that of reference datasets, suggesting that Mol-CADiff captures the underlying physicochemical manifold of real-world drugs.

### Target-specific design and potential:

We also investigate the potential of our framework to support target-specific design, aiming to support lead optimization. To explore this, we assessed the generation of potential inhibitors for the Epidermal Growth Factor Receptor. Mol-CADiff produced molecules with fingerprint similarity distributions comparable to those of known EGFR inhibitors, suggesting that the model captures target-relevant pharmacophore features. These findings indicate that our approach holds promise for generating candidates that are not just chemically valid but also biologically plausible.

### Limitations and future directions

Despite its strengths, Mol-CADiff presents several limitations that warrant attention. First, although Mol-CADiff generates high-quality small molecules, its performance may still be dependent on the scale and diversity of the training data. If the dataset used to learn the latent space is limited to specific chemical families, the synthetic molecules produced may inherit these biases, potentially limiting applicability to novel protein targets or macrocycles. Additionally, while Mol-CADiff demonstrates strong performance on standard benchmarks, its generalization capabilities may diminish when faced with very large molecules or complex biologics that differ significantly from the training distribution. Furthermore, although our model generates molecules with high Novelty and Diversity, it does not currently output an explicit retrosynthetic pathway. This means that while the molecules are theoretically synthesizable, their practical realization in the wet lab may still require significant expert intervention. Addressing these limitations in future iterations of Mol-CADiff would be crucial for broadening its applicability to complex therapeutic modalities.

To address these limitations, future research on Mol-CADiff can progress in multiple directions. A key area is extending the causal attention framework to handle larger molecular systems, such as peptides or protein-ligand complexes. This could involve refining the tokenization strategy to capture hierarchical structures or incorporating 3D geometric constraints directly into the diffusion process. Additionally, using multi-objective optimization techniques could help Mol-CADiff generalize more effectively when balancing conflicting design criteria, such as maximizing potency while minimizing toxicity. Another important direction is integrating retrosynthesis prediction directly into the generation loop to ensure that every generated molecule comes with a viable synthesis recipe. This would ensure more reliable utility in real-world laboratory settings. Furthermore, integrating feedback from high-throughput screening assays into the training process could increase its clinical relevance, aligning outputs more closely with experimental outcomes. These research directions could enhance Mol-CADiff’s adaptability and effectiveness across diverse stages of the drug discovery pipeline.

Mol-CADiff is a robust molecular generation method that seamlessly integrates causal autoregression with diffusion modeling. It enhances the generation of novel, diverse, and property-aligned molecules, boosting the efficiency of text-conditional design. Furthermore, it surpasses alternative methods in capturing the complex dependencies required for realistic drug discovery applications.

## Methods

We consider a molecule represented as a graph *G* along with its corresponding textual description *C* as a condition. Our goal is to develop a causality-aware auto-regressive framework that generates novel, diverse, and chemically valid molecules while preserving the properties described in the given condition. Formally, a molecule is represented as *G* = (*V*, *A*), where *V* is the set of *n* nodes, and $A\in {{\mathbb{R}}}^{n\times n\times a}$ is the adjacency matrix encoding edge features. Each entry in *A* is one-hot encoded, where each distinct value corresponds to a specific bond type.

### Pretraining stages

To enable efficient diffusion modeling, we first transform molecular graphs and their corresponding textual descriptions into a compact latent space. This reduces computational complexity by operating on fewer tokens rather than the full graph-text representation. We employ two pretraining strategies: contrastive learning for aligning graph and text representations and encoder-decoder training for structured graph reconstruction.

First, we utilize a pretrained Graph Encoder *G*_E_ and Text Encoder *C*_E_, both optimized using contrastive learning, for example, GIN^61^ as *G*_E_ and Sci-BERT^62^ as *C*_E_. The latent representations, *L*_g_ and *L*_c_, extracted from *G*_E_ and *C*_E_, respectively, are aligned via contrastive learning on a dataset of molecule-text pairs^44^, enforcing similarity between corresponding pairs while distinguishing unrelated ones.

Next, we train the Graph Encoder-Decoder pair (*G*_E_, *G*_D_) in an autoencoder-style manner to reconstruct molecular structures. The Graph Decoder *G*_D_ (e.g., HierVAE^16^) reconstructs the molecular graph $\hat{G}$ from the latent representation *L*_g_, optimizing the negative Evidence Lower Bound (ELBO)^43^. These pretraining steps reduce the complexity of diffusion modeling by operating in a structured latent space rather than raw molecular graphs and text, thereby improving computational efficiency.

### Integrating autoregression with diffusion

**Fig. 7:**
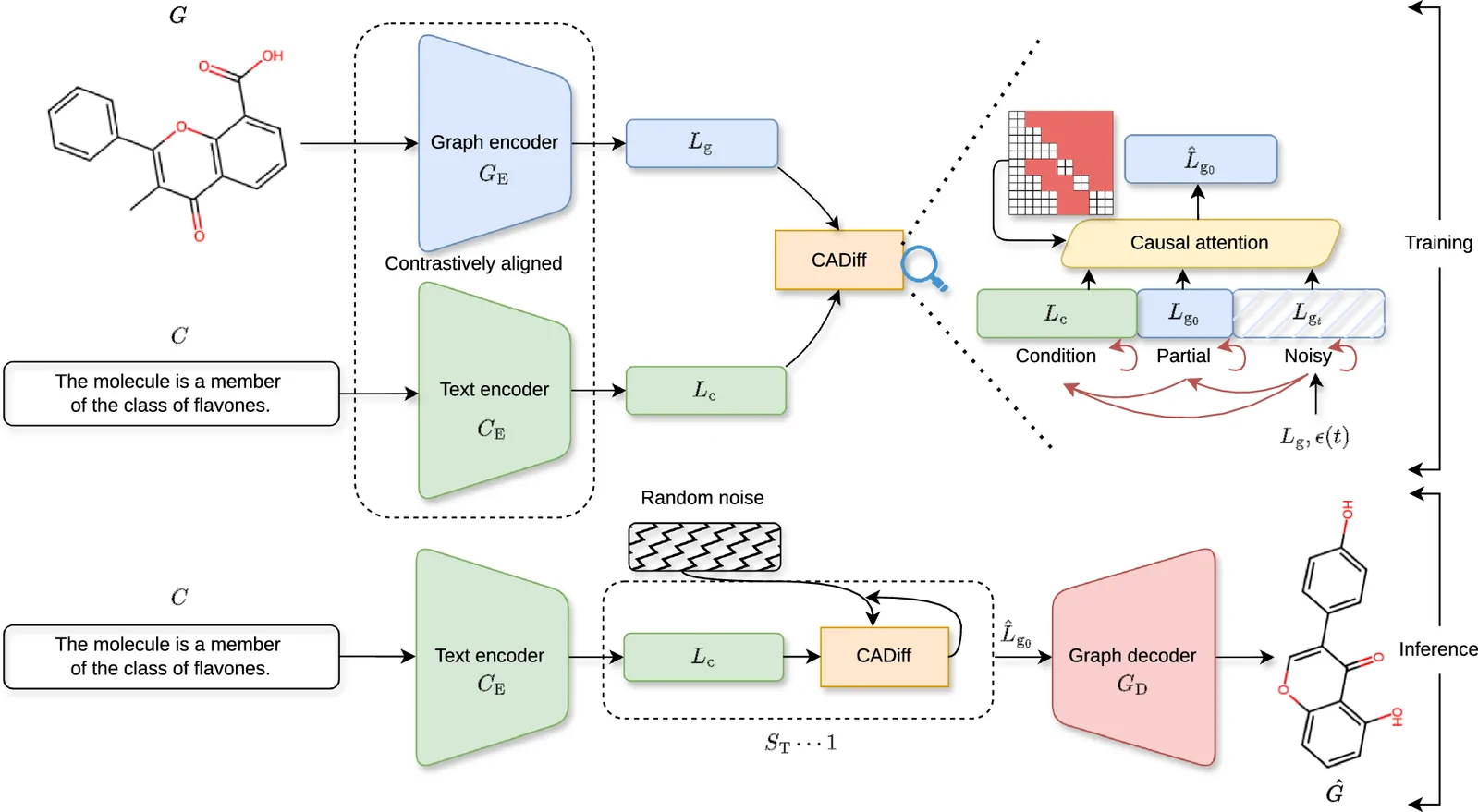
Schematic of the Mol-CADiff framework. Encoders *G*_E_ and *C*_E_ map molecular graphs (*G*) and text instructions (*C*) to their respective latents (*L*_g_ and *L*_c_). The CADiff module integrates autoregressive generation with diffusion, ensuring causal dependencies through multimodal attention. During inference, the text instructions *C* guide iterative denoising to produce the predicted graph latent ${\hat{L}}_{{{{\rm{g}}}}_{0}}$, which is subsequently decoded by the decoder *G*_D_ into the final molecule. Blue indicates graph-related encoders and latent representations, green indicates text-related encoders and condition latents, orange indicates the CADiff and causal attention modules, pink indicates the graph decoder, red arrows indicate causal attention dependencies, black arrows indicate information flow, and molecular atom colors follow standard chemical conventions.

To alleviate diffusion modeling and generate diverse, novel, and chemically valid molecules, we introduce an autoregressive causal dependency as demonstrated in Fig. 7 under the CADiff module. This module requires three key latent representations: the conditional text-based latent representation *L*_c_, the partial clean latent representation of the molecular graph ${L}_{{{{\rm{g}}}}_{0}}$, and the noisy graph latent representation ${L}_{{{{\rm{g}}}}_{t}}$ at diffusion timestep *t*. For consistency, we denote the unperturbed (clean) latent representation *L*_g_ as ${L}_{{{{\rm{g}}}}_{0}}$, corresponding to diffusion timestep *t* = 0. The forward diffusion process progressively perturbs ${L}_{{{{\rm{g}}}}_{0}}$ by adding Gaussian noise, producing a sequence of noisy latents $\{{L}_{{{{\rm{g}}}}_{1}},{L}_{{{{\rm{g}}}}_{2}},...,{L}_{{{{\rm{g}}}}_{T}}\}$. This follows a Markovian diffusion process, where at each step *t*, the latent representation is perturbed based on a predefined variance schedule:

1 $$q({L}_{{{{\rm{g}}}}_{t}}| {L}_{{{{\rm{g}}}}_{t-1}})={{\mathcal{N}}}\left({L}_{{{{\rm{g}}}}_{t}};\sqrt{1-{\beta }_{t}}{L}_{{{{\rm{g}}}}_{t-1}},{\beta }_{t}I\right),$$

where *β*_*t*_ is the variance schedule at timestep *t*, and *I* is the identity matrix. The variance schedule ${\{{\beta }_{t}\}}_{t=1}^{T}$ is a monotonically increasing sequence, ensuring a gradual injection of noise. The full forward diffusion process, allowing direct computation of ${L}_{{{{\rm{g}}}}_{t}}$ from ${L}_{{{{\rm{g}}}}_{0}}$, is given by:

2 $$q({L}_{{{{\rm{g}}}}_{t}}| {L}_{{{{\rm{g}}}}_{0}})={{\mathcal{N}}}\left({L}_{{{{\rm{g}}}}_{t}};\sqrt{{\bar{\alpha }}_{t}}{L}_{{{{\rm{g}}}}_{0}},(1-{\bar{\alpha }}_{t})I\right),$$

3 $${L}_{{{{\rm{g}}}}_{t}}=\sqrt{{\bar{\alpha }}_{t}}{L}_{{{{\rm{g}}}}_{0}}+\sqrt{1-{\bar{\alpha }}_{t}}\epsilon,\quad \,{{\mbox{where}}}\,\epsilon \sim {{\mathcal{N}}}(0,I),$$

where ${\bar{\alpha }}_{t}={\prod }_{s=1}^{t}(1-{\beta }_{s})$ is the cumulative product of noise reduction terms. The full joint probability distribution over all diffusion steps is then factorized as:

4 $$q({L}_{{{{\rm{g}}}}_{0:T}})=q({L}_{{{{\rm{g}}}}_{0}})\mathop{\prod }_{t=1}^{T}q({L}_{{{{\rm{g}}}}_{t}}| {L}_{{{{\rm{g}}}}_{t-1}}).$$

This formulation effectively models the progressive noise injection process while preserving structural dependencies essential for molecular generation. While diffusion models are well-suited for image generation and denoising tasks, they inherently lack the ability to capture dependencies in a causal autoregressive manner.

To address this, we replace standard attention with a causal attention mechanism and adopt an autoregressive token generation strategy, which enables diffusion and autoregression to operate jointly. Formally, let $X=[{L}_{{{\rm{c}}}},\,{L}_{{{{\rm{g}}}}_{0,{\kappa }_{1:s-1}}},\,{L}_{{{{\rm{g}}}}_{t,{\kappa }_{s}}}]$ denote the concatenated sequence at AR step *s*, consisting of conditional text tokens *L*_c_, the clean latent tokens from previous AR steps ${L}_{{{{\rm{g}}}}_{0,{\kappa }_{1:s-1}}}$, and the noisy tokens for the current AR step ${L}_{{{{\rm{g}}}}_{t,{\kappa }_{s}}}$. Following standard attention^70^, we obtain query, key, and value matrices through learned linear projections,

5 $$Q=X{W}_{{{\rm{Q}}}},\qquad K=X{W}_{{{\rm{K}}}},\qquad V=X{W}_{{{\rm{V}}}}.$$

A standard attention layer computes

6 $${{\rm{Attn}}}(Q,K,V)={{\rm{softmax}}}\,\left(\frac{Q{K}^{\top }}{\sqrt{d}}\right)V.$$

But this allows every token to attend to every other token. To enforce correct autoregressive dependencies, we introduce a binary causal mask *M* ∈ {0, 1}^*N *× *N*^ that restricts information flow, where *N* denotes the total number of tokens in the sequence *X*. The masked attention becomes

7 $${{\rm{Attn}}}(Q,K,V;M)={{\rm{softmax}}}\,\left(\frac{Q{K}^{\top }}{\sqrt{d}}+A\right)V,$$

where

8 $${A}_{ij}=\left\{\begin{array}{ll}-\infty,\quad &\,{{\mbox{if}}}\,{M}_{ij}=1,\hfill \\ 0,\hfill \quad &\,{\mbox{otherwise}}\,,\end{array}\right.$$

ensuring that masked positions receive zero probability after the softmax. The mask is constructed so that: (i) noisy tokens at AR step *s* cannot access future latent tokens, (ii) clean tokens from earlier AR steps may guide the denoising of the current step, and (iii) conditional text tokens *L*_c_ remain visible to all positions. This prevents information leakage from future diffusion or AR steps and allows conditional tokens to act as global context while previously denoised tokens steer the current denoising process.

Unlike image generation, which relies on spatial dependencies within patches and class labels, molecular graph data involves high-dimensional continuous values and complex relational structures. Our approach effectively addresses these challenges through four key components: (1) a conditional causality-aware attention mechanism that captures continuous expression levels from text embeddings rather than discrete class labels; (2) a token-based processing system, where compressed graph and text representations function as tokens; (3) preservation of token ordering across multiple modalities rather than shuffling them; and (4) a denoising strategy that directly predicts the clean version of tokens instead of modeling added noise, diverging from CausalFusion^71^.

This integration of autoregression and diffusion enables the model to capture structural dependencies while effectively generating novel and diverse molecular graphs. More formally, the integration of AR with diffusion can be represented by:

9 $$q({L}_{{{{\rm{g}}}}_{0:T,{\kappa }_{s}}}| {L}_{{{{\rm{g}}}}_{0,{\kappa }_{1:s-1}}})=q({L}_{{{{\rm{g}}}}_{0,{\kappa }_{s}}}){\prod }_{t=1}^{T}q({L}_{{{{\rm{g}}}}_{t,{\kappa }_{s}}}| {L}_{{{{\rm{g}}}}_{t-1,{\kappa }_{s}}},{L}_{{{{\rm{g}}}}_{0,{\kappa }_{1:s-1}}}).$$

In Eq. (9), *S* represents the number of AR steps, where *s* ∈ [1, *S*], and *κ*_*s*_ denotes the index set of tokens processed at the *s*^th^ AR step. The number of tokens in each AR step is given by ∣*κ*_*s*_∣, and only the tokens indexed by *κ*_*s*_ are processed during that step. Here, ${L}_{{{{\rm{g}}}}_{t,{\kappa }_{s}}}$ represents the dual-factorized graph tokens at the *s*^th^ AR step and *t*^th^ diffusion step. During training, our objective is to approximate ${p}_{\theta }({L}_{{{{\rm{g}}}}_{t-1},{\kappa }_{s}}| {L}_{{{{\rm{g}}}}_{t,{\kappa }_{s}}},{L}_{{{{\rm{g}}}}_{0,{\kappa }_{1:s-1}}})$ for all *t* and *s*. This estimation also requires the partial clean latent representation ${L}_{{{{\rm{g}}}}_{0,{\kappa }_{1:s-1}}}$ with the noisy tokens of the current AR step.

This structure allows the model to leverage information from previous AR steps, improving the denoising of the current tokens. A generalized causal attention mask ensures that ${L}_{{{{\rm{g}}}}_{0,{\kappa }_{1:s-1}}}$ does not have access to ${L}_{{{{\rm{g}}}}_{t,{\kappa }_{s}}}$, enforcing proper sequential dependencies. We illustrate an example of this causal attention mechanism in Figs. 6, 7. Effectively, this can be interpreted as a next-token prediction task, where each token attends to previously generated tokens while simultaneously being denoised through diffusion. By incorporating the causal attention mask, we maintain structured dependencies both within the same modality (intra-modal) and across different modalities (inter-modal).

### Autoregressive steps formulation

We generate AR steps (as) using an algorithm (see Box 1), which takes the sample length *l* and decay factor *γ* as inputs. When *γ* = 1.0, the AR step count is uniformly sampled, leading to an unbiased distribution of step sizes. For *γ* < 1.0, smaller step sizes are favored at the beginning due to the exponential decay, resulting in progressively larger steps later in the sequence. This behavior affects how information is structured across autoregressive steps, and we further analyze its effect in the ablation study (Fig. 5a).

Box 1 Algorithm to generate AR steps **Input:** *l* (length), *γ* (decay)

1: *I* ← Uniform([1, *l*]) if *γ* = 1.0, else sample from [1, *l*] with *p*_*i*_ = *b* ⋅ *γ*^*i*^ for *i* ∈ [0, *l* − 1], where $b=\frac{1-\gamma }{1-{\gamma }^{l}}$

2: cm ← [0] ∪ sort(random sample from[1, *l*) of size *I* − 1) ∪ [*l*]

3: as ← [cm[*i* + 1] − cm[*i*]∣*i* ∈ [0, *I* − 1]]

**return** as, cm

### Inferencing from instruction

During the inference (test) phase, molecular generation depends on whether the task is conditional or unconditional. For conditional generation, we use only the provided instruction *C* to guide the process, whereas for unconditional generation, no such instruction is required. While Fig. 7 illustrates the framework for conditional generation, our method seamlessly extends to unconditional generation by omitting the conditioning information from *L*_c_. During inference, we initialize the process with random noise and iteratively refine it over sampling timesteps, from *S*_T_ down to 1. The CADiff module is responsible for this iterative denoising, progressively reconstructing the latent representation. Once the denoised latent representation ${\hat{L}}_{{{{\rm{g}}}}_{0}}$ is obtained, it is passed through the Graph Decoder *G*_D_, which generates the final molecular structure $\hat{G}$. This approach ensures a structured, stepwise refinement of molecular representations, leading to high-quality, valid, and diverse molecular samples.

#### Autoregressive behavior at inference

During inference, our model generates latent tokens sequentially. At each step, it maintains a key–value (KV) cache that stores only the representations of tokens generated so far. When producing the next token, the attention mechanism retrieves information exclusively from this cache. Because future tokens have not yet been generated and thus do not exist in the cache, the model is inherently restricted to attending only to past information. This structure preserves the autoregressive property without requiring an explicit causal mask at inference time. In contrast, during training, clean latent tokens from earlier autoregressive segments are provided alongside the noised tokens of the current segment, making a causal mask necessary to prevent access to future positions. This separation ensures that the model learns correct dependency patterns during training while supporting efficient decoder-style generation during inference.

### Reporting summary

Further information on research design is available in the Nature Portfolio Reporting Summary linked to this article.

## Supplementary information


Supplementary Information
Description of Additional Supplementary Files
Supplementary data 1 (Unprocessed raw data)
Reporting Summary
Transparent Peer Review file


## Source data


Source Data


## Data Availability

The data that support the findings of this study are available from the corresponding authors upon request. The datasets used in this study (ChEBI-20, PubChem, PCDes, and MoMu) are publicly available and can be accessed via the repositories linked to the refs. ^39–41,43,44^. Unprocessed raw data are provided as Supplementary Data 1. Source data are provided with this paper.

## Code availability

The source code of Mol-CADiff is available from Zenodo^72^ and from the corresponding author upon request.
