# Supplementary material for: Mol-CADiff: text-conditional molecule generation via causality-aware autoregressive diffusion
Source: Nat Commun. 2026 Jul 29;17:9190. doi: 10.1038/s41467-026-75702-5 (PMC13522512; doi:10.1038/s41467-026-75702-5)
Supplement: Supplementary file 1 [file 41467_2026_75702_MOESM1_ESM.pdf]

Supplementary Information

Mol-CADiff: text-conditional molecule generation via causality-aware autoregressive diffusion

Supplementary Note 1. Overview of supplementary information

This supplementary document provides additional details and experimental results that complement our main paper. We include a detailed algorithm for the autoregressive causal attention mask, extended qualitative results for conditionally and unconditionally generated molecules, and additional case studies comparing Mol-CADiff with other baselines. These materials further demonstrate the effectiveness of our proposed method. To distinguish materials in this supplement from the main text, we add the prefix "Supplementary" before all Figures, Tables, and Boxes.

Supplementary Note 2. Dataset statistics

Supplementary Table 1. Dataset statistics. Number of samples across training, validation, and test sets for the four benchmark datasets.

Dataset	Training	Validation	Test
ChEBI-20	15,409	1,971	1,965
PubChem	6,912	571	1,162
PCDes	7,474	1,051	2,136
MoMu	7,474	1,051	4,554

Supplementary Note 3. Autoregressive causal mask

Our framework integrates a causal attention mechanism to model dependencies in an autoregressive manner, ensuring structured molecule generation. This section provides a detailed algorithm for generating the causal attention mask, which controls the directional flow of information during training. The algorithm enforces appropriate token dependencies, allowing conditional text tokens and partially cleaned previously denoised graph latents to guide the denoising process effectively. The full procedure is detailed in an algorithm (see Supplementary Box 1).

Supplementary Box 1. Algorithm for autoregressive causal attention mask.

Input: l (sample length), cl (conditional length), as (split sizes), cm (cumulative sum of split sizes)
Output: Causal attention mask M

$v \leftarrow l - \text{as}[-1]$, $\text{ctx} \leftarrow \text{cl} + v$, $\text{seq} \leftarrow \text{ctx} + l$
Initialize $M \in \mathbb{R}^{\text{seq}\times\text{seq}}$ with ones
$M[:, :\text{cl}] \leftarrow 0$
$T_1 \leftarrow 1^{v\times v}$, $T_2 \leftarrow 1^{l\times v}$, $T_3 \leftarrow 1^{l\times l}$
for $i = 0$ to $|\text{as}| - 2$ **do**
  $T_1[\text{cm}[i] : \text{cm}[i+1], 0 : \text{cm}[i+1]] \leftarrow 0$
  $T_2[\text{cm}[i+1] : \text{cm}[i+2], 0 : \text{cm}[i+1]] \leftarrow 0$
end for
for $i = 0$ to $|\text{as}| - 1$ **do**
  $T_3[\text{cm}[i] : \text{cm}[i+1], \text{cm}[i] : \text{cm}[i+1]] \leftarrow 0$
end for
$M[\text{cl} : \text{ctx}, \text{cl} : \text{ctx}] \leftarrow T_1$, $M[\text{ctx} :, \text{cl} : \text{ctx}] \leftarrow T_2$, $M[\text{ctx} :, \text{ctx} :] \leftarrow T_3$
return M

Supplementary Note 4. Conditionally generated samples

To further validate the effectiveness of Mol-CADiff in text-conditional molecular generation, we provide additional samples generated based on prompts from the ChEBI-20 test set. These descriptions were unseen during training, ensuring a rigorous

assessment of the model's generalization capabilities. As shown in Supplementary Figure 1, the generated molecules maintain high fidelity to the textual conditions while preserving strict structural Validity. These results highlight the ability of our framework to translate complex chemical semantics into salient molecular structures in a chemically meaningful manner.

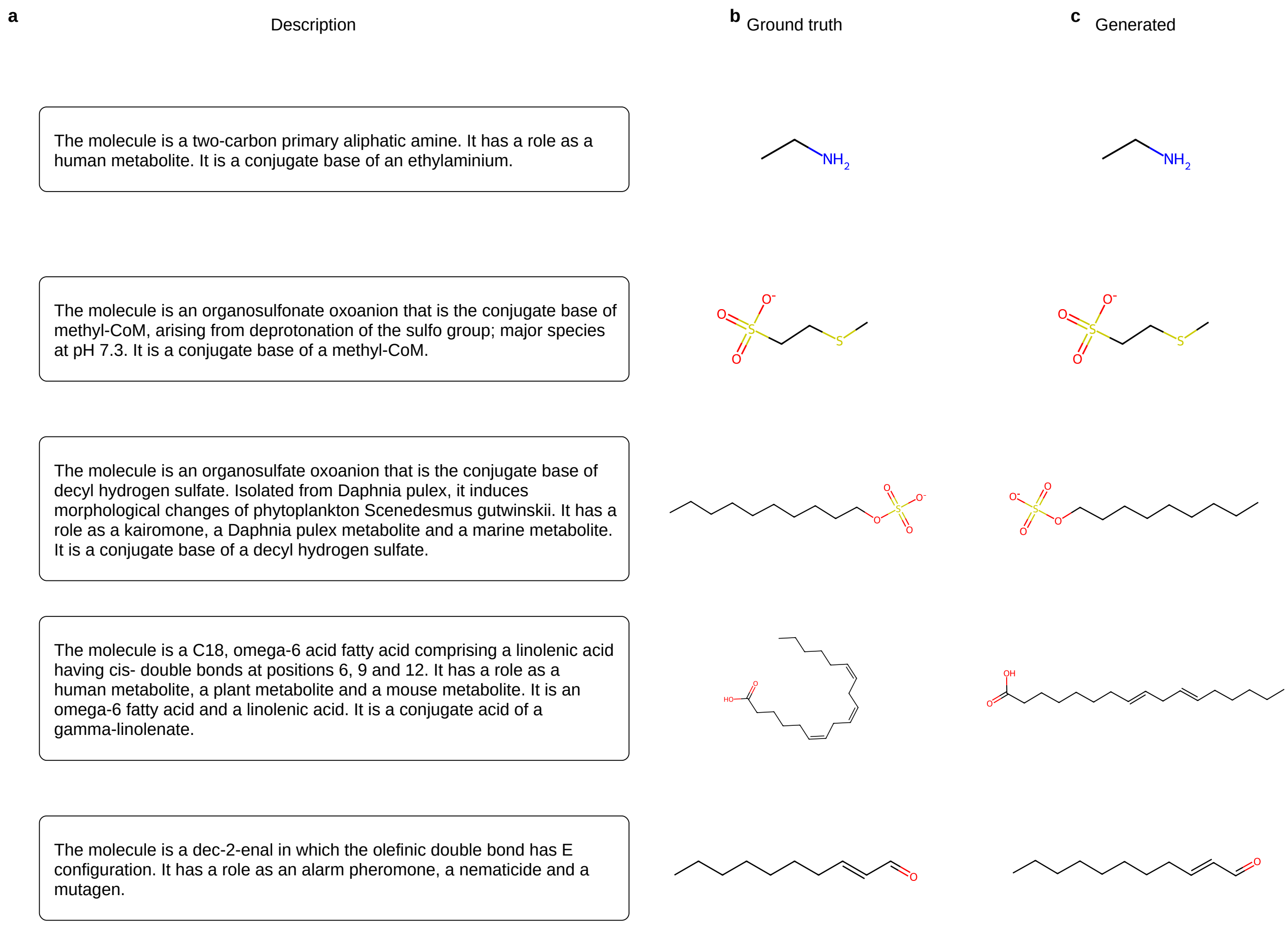

Supplementary Figure 1. Qualitative examples of text-conditioned molecule generation. Representative molecules generated from the ChEBI-20 test set using provided text prompts. **a**, Text descriptions used as molecular generation prompts. **b**, Ground truth molecules corresponding to the prompts. **c**, Generated molecular structures. The generated structures demonstrate high alignment with the ground-truth molecules and accurately reflect the chemical properties described in the text.

Supplementary Note 5. Unconditionally generated samples

In addition to conditional generation, Mol-CADiff is capable of generating molecules without textual prompts by sampling directly from the learned latent distribution. Supplementary Figure 2 presents a variety of valid and diverse molecules generated in this unconstrained setting, demonstrating the flexibility of our framework in exploring the broader chemical manifold. These results confirm that Mol-CADiff effectively captures the underlying distribution of molecular structures, enabling the generation of chemically plausible and novel candidates even in the absence of textual guidance.

Supplementary Note 6. Additional case studies

To further analyze the performance of Mol-CADiff, we compare its generated molecules with those from 3M-Diffusion and MolT5-large across diverse chemical property conditions. The following studies provide a qualitative assessment of the model's ability to steer molecular generation based on specific semantic instructions. Each row in the associated tables contains molecules generated by different models, along with their respective Quantitative Estimate of Drug-likeness or Similarity scores.

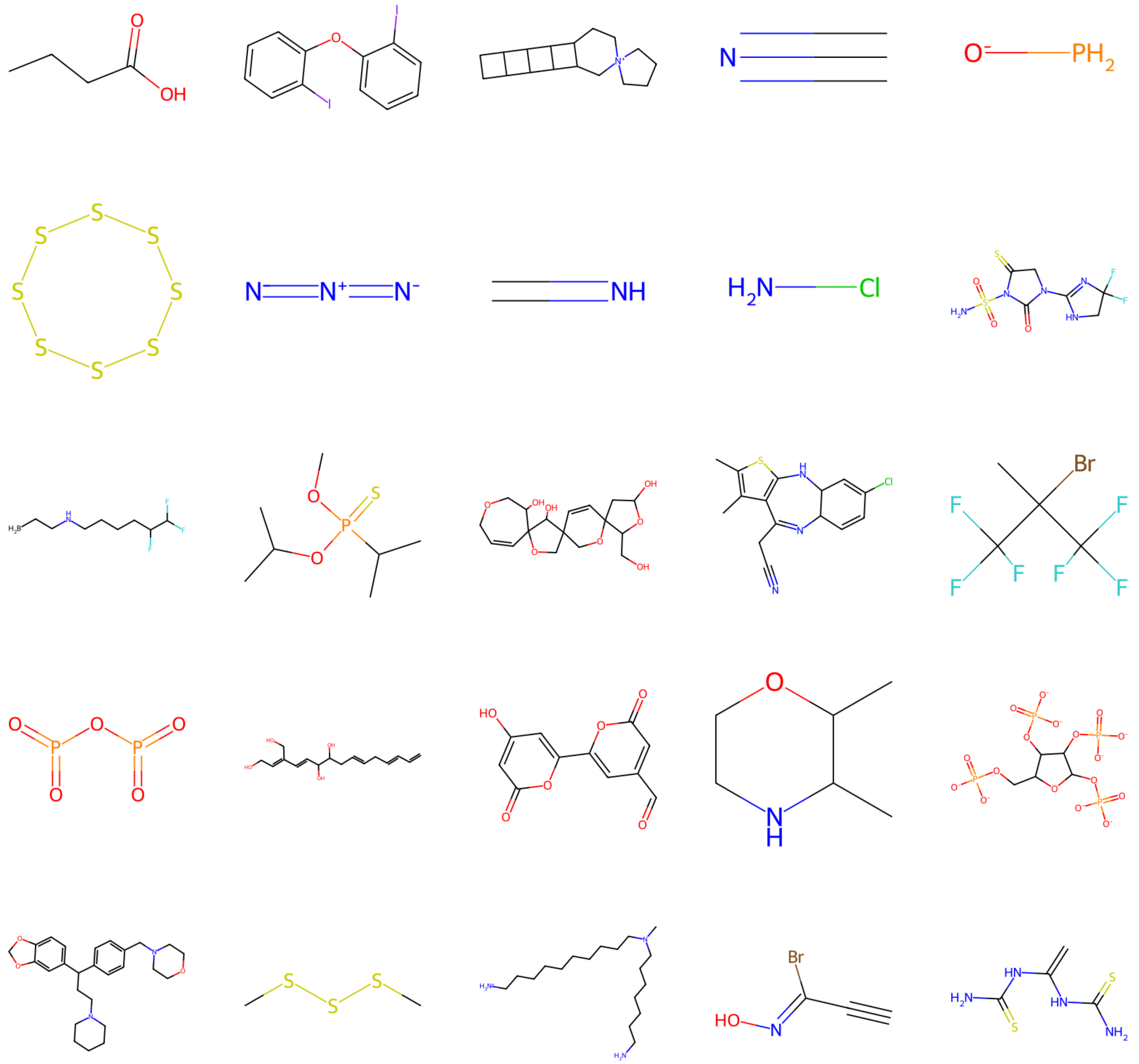

Supplementary Figure 2. Unconditionally generated samples. A random selection of generated molecules demonstrating high chemical Validity and structural Diversity.

Drug-likeness Study: Supplementary Figure 3 presents molecules generated under the condition: "This molecule is like a drug." Mol-CADiff consistently achieves higher QED values compared to baseline models, demonstrating stronger alignment with desired pharmacological properties.

Non-Drug Molecule Study: Conversely, Supplementary Figure 4 illustrates molecules generated under the opposite constraint: "This molecule is not like a drug." In this setting, Mol-CADiff exhibits significantly lower QED values, confirming that the framework effectively captures the underlying property gradient.

Functional Group Matching: Supplementary Figure 5 showcases molecules generated under a specific structural condition: "The molecule is an anthocyanidin cation." Similarity scores relative to reference molecules indicate that Mol-CADiff achieves significantly higher structural fidelity and functional group alignment than the baselines.

These case studies further validate that Mol-CADiff does not merely produce generic valid structures, but effectively captures the nuanced chemical semantics required for precise, text-conditional molecular design.

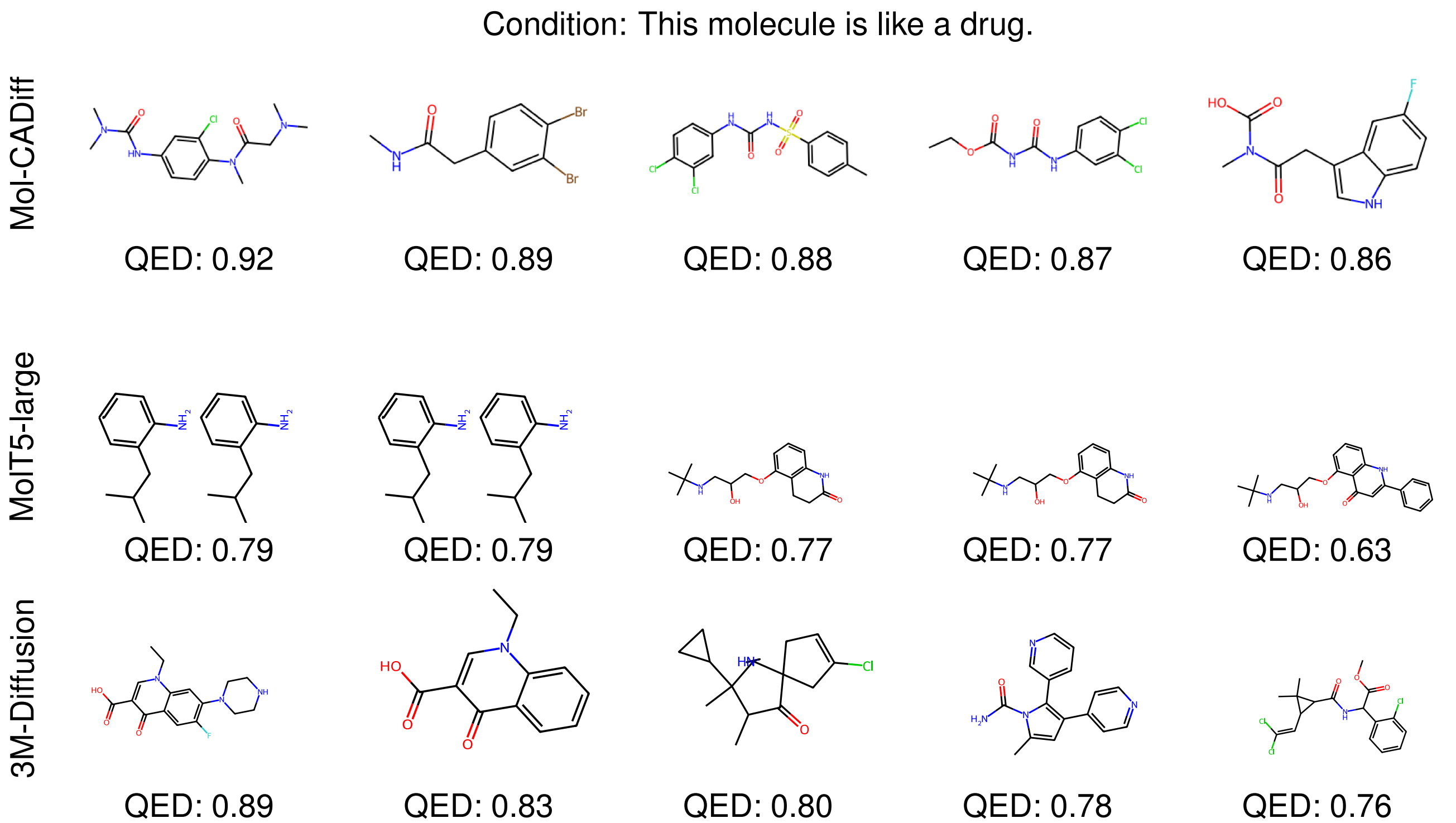

Supplementary Figure 3. Comparative study of drug-like molecule generation. Comparison of molecules generated by Mol-CADiff, MolT5-large, and 3M-Diffusion under the text condition "This molecule is like a drug." For each model, the top five samples are displayed with their Quantitative Estimate of Drug-likeness scores. Mol-CADiff generates structures with higher QED values across diverse chemical scaffolds, outperforming the baselines in translating abstract text instructions into high-quality molecular candidates.

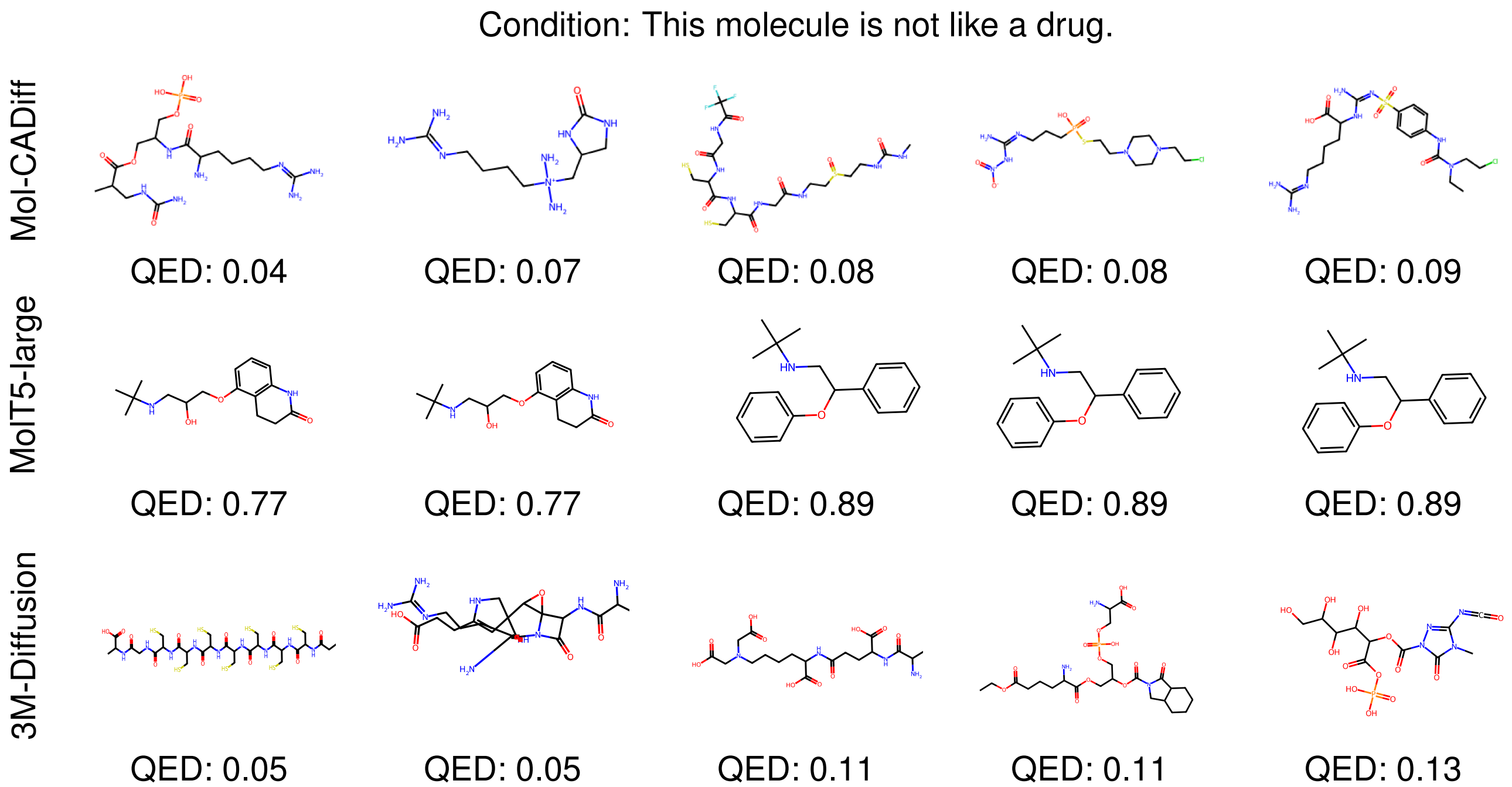

Supplementary Figure 4. Comparative study of non-drug-like molecule generation. Comparison of molecules generated by Mol-CADiff, MolT5-large, and 3M-Diffusion under the text condition "This molecule is not like a drug." For each model, the top five samples are displayed. In this context, lower QED values indicate higher alignment with the negative drug-likeness instruction. Mol-CADiff successfully generates non-drug-like structures (QED < 0.1) in response to the prompt, whereas some baselines (e.g., MolT5-large) continue to generate high-QED drug-like molecules, demonstrating the superior instruction-following capabilities of our framework.

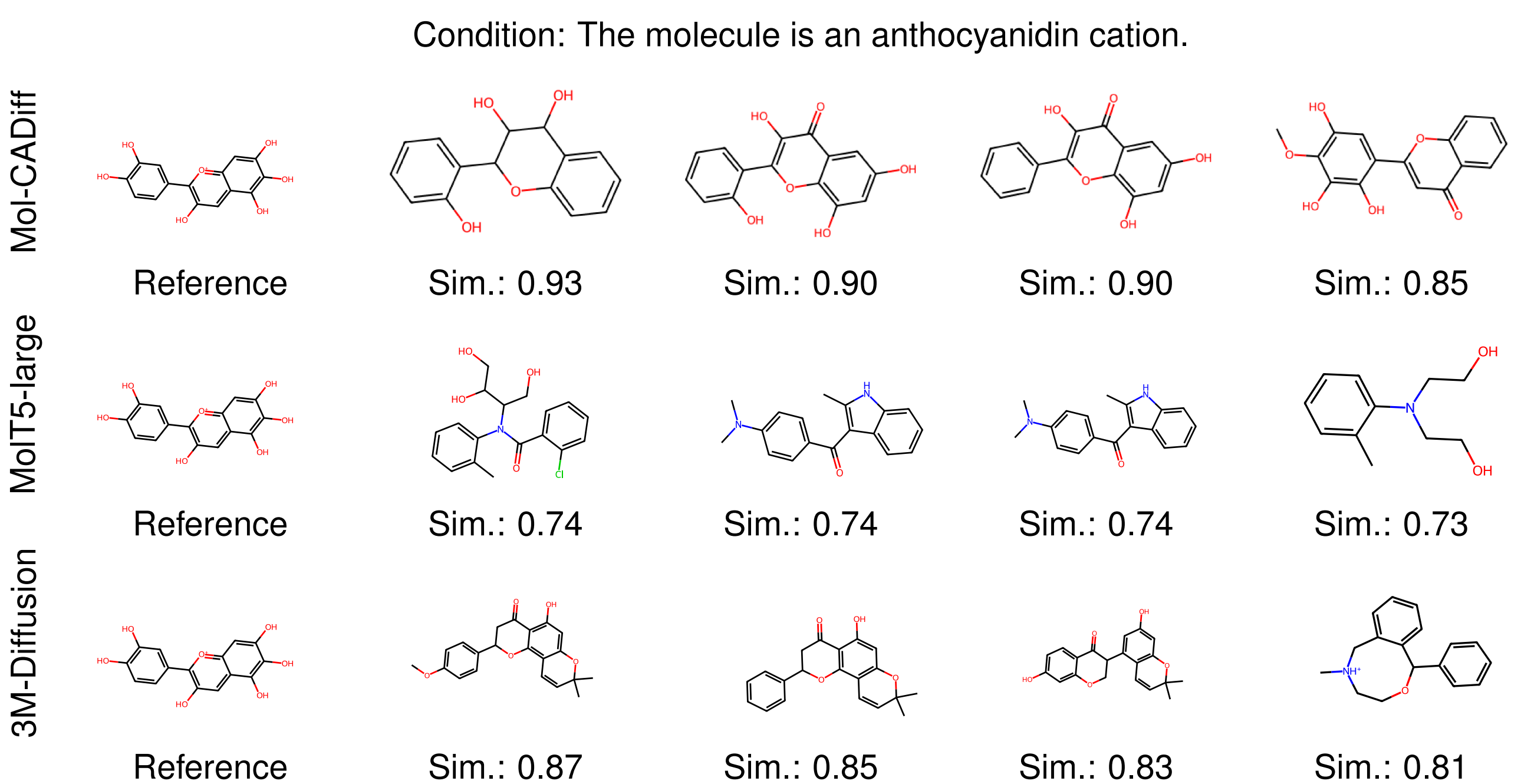

Supplementary Figure 5. Case study of anthocyanidin cation generation. Comparison of molecules generated by Mol-CADiff, MolT5-large, and 3M-Diffusion conditioned on the prompt "The molecule is an anthocyanidin cation." The first column displays the reference (ground-truth structure). The subsequent four columns show generated samples with their corresponding Similarity (Sim.) to the reference. Mol-CADiff demonstrates superior structural fidelity, achieving higher Similarity scores compared to the baselines.
