# Supplementary material for: Mol-CADiff: text-conditional molecule generation via causality-aware autoregressive diffusion
Source: Nat Commun. 2026 Jul 29;17:9190. doi: 10.1038/s41467-026-75702-5 (PMC13522512; doi:10.1038/s41467-026-75702-5)
Supplement: Supplementary file 2 [file 41467_2026_75702_MOESM2_ESM.pdf]

Description of Additional Supplementary Files

File Name: Supplementary Data 1

Description: Unprocessed raw data
