# Supplementary material for: Mol-CADiff: text-conditional molecule generation via causality-aware autoregressive diffusion
Source: Nat Commun. 2026 Jul 29;17:9190. doi: 10.1038/s41467-026-75702-5 (PMC13522512; doi:10.1038/s41467-026-75702-5)
Supplement: Supplementary file 3 [file 41467_2026_75702_MOESM4_ESM.pdf]

nature portfolio

Corresponding author(s): Qiang Cheng

Last updated by author(s): Jul 1, 2026

Reporting Summary

Nature Portfolio wishes to improve the reproducibility of the work that we publish. This form provides structure for consistency and transparency in reporting. For further information on Nature Portfolio policies, see our Editorial Policies and the Editorial Policy Checklist.

Statistics

For all statistical analyses, confirm that the following items are present in the figure legend, table legend, main text, or Methods section.

n/a	Confirmed	
☐	☒	The exact sample size (*n*) for each experimental group/condition, given as a discrete number and unit of measurement
☐	☒	A statement on whether measurements were taken from distinct samples or whether the same sample was measured repeatedly
☐	☒	The statistical test(s) used AND whether they are one- or two-sided<br>*Only common tests should be described solely by name; describe more complex techniques in the Methods section.*
☒	☐	A description of all covariates tested
☐	☒	A description of any assumptions or corrections, such as tests of normality and adjustment for multiple comparisons
☐	☒	A full description of the statistical parameters including central tendency (e.g. means) or other basic estimates (e.g. regression coefficient) AND variation (e.g. standard deviation) or associated estimates of uncertainty (e.g. confidence intervals)
☐	☒	For null hypothesis testing, the test statistic (e.g. *F*, *t*, *r*) with confidence intervals, effect sizes, degrees of freedom and *P* value noted<br>*Give P values as exact values whenever suitable.*
☒	☐	For Bayesian analysis, information on the choice of priors and Markov chain Monte Carlo settings
☒	☐	For hierarchical and complex designs, identification of the appropriate level for tests and full reporting of outcomes
☒	☐	Estimates of effect sizes (e.g. Cohen's *d*, Pearson's *r*), indicating how they were calculated

Our web collection on statistics for biologists contains articles on many of the points above.

Software and code

Policy information about availability of computer code

Data collection	No new experimental data were collected, and no data collection software was used.
Data analysis	Data analysis was performed using Python 3.8.16, PyTorch 2.0.0, PyTorch Geometric 2.3.0, CUDA 11.7, RDKit 2023.9.3, NumPy 1.24.3, pandas 1.5.3, scikit-learn 1.2.2, SciPy 1.10.1, Matplotlib 3.7.3, GuacaMol 0.5.5, Transformers 4.26.1, and custom Mol-CADiff scripts. More detailed packages and versions are provided in the Custom code, available at https://github.com/Atik-Ahamed/Mol-CADiff and archived at https://zenodo.org/records/21108095.

For manuscripts utilizing custom algorithms or software that are central to the research but not yet described in published literature, software must be made available to editors and reviewers. We strongly encourage code deposition in a community repository (e.g. GitHub). See the Nature Portfolio guidelines for submitting code & software for further information.

Data

Policy information about availability of data

All manuscripts must include a data availability statement. This statement should provide the following information, where applicable:

- Accession codes, unique identifiers, or web links for publicly available datasets
- A description of any restrictions on data availability
- For clinical datasets or third party data, please ensure that the statement adheres to our policy

The data that support the findings of this study are available from the corresponding authors upon request. The datasets used in this study (ChEBI-20, PubChem,

PCDes, and MoMu) are publicly available and can be accessed via the repositories linked refs. 39–41, 43, 44. Unprocessed raw data are provided as Supplementary Data 2. Source data are provided with this paper.

Research involving human participants, their data, or biological material

Policy information about studies with human participants or human data. See also policy information about sex, gender (identity/presentation), and sexual orientation and race, ethnicity and racism.

Reporting on sex and gender	Not applicable. This study uses publicly available datasets and does not involve human participants, human data, or biological material.
Reporting on race, ethnicity, or other socially relevant groupings	Not applicable. This study uses publicly available datasets and does not involve human participants, human data, or biological material.
Population characteristics	Not applicable. This study uses publicly available datasets and does not involve human participants, human data, or biological material.
Recruitment	Not applicable. This study uses publicly available datasets and does not involve human participants, human data, or biological material.
Ethics oversight	Not applicable. This study uses publicly available datasets and does not involve human participants, human data, or biological material.

Note that full information on the approval of the study protocol must also be provided in the manuscript.

Field-specific reporting

Please select the one below that is the best fit for your research. If you are not sure, read the appropriate sections before making your selection.

☒ Life sciences ☐ Behavioural & social sciences ☐ Ecological, evolutionary & environmental sciences

For a reference copy of the document with all sections, see nature.com/documents/nr-reporting-summary-flat.pdf

Life sciences study design

All studies must disclose on these points even when the disclosure is negative.

Sample size	We used publicly available molecular benchmark datasets. The number and choice of datasets were determined based on prior published molecular generation studies and were kept the same to ensure fair comparison with existing methods. These datasets are sufficient because they cover both text-guided conditional and unconditional molecule generation tasks across multiple benchmark settings.
Data exclusions	No datasets were excluded. For consistency with prior molecular generation benchmarks, analyses were performed using prior published processing techniques. This criterion was predefined and applied consistently across the relevant datasets.
Replication	Computational experiments were repeated where stochastic variability was assessed. Analyses with error bars were performed. Other analyses used predefined benchmark test sets or representative runs as indicated in the figure legends and Source Data.
Randomization	Samples were allocated according to the predefined training, validation, and test splits used in the public benchmark datasets and prior studies. No additional random allocation into biological or clinical experimental groups was performed.
Blinding	Blinding was not relevant to this computational study. All experiments were performed on predefined public datasets. No human participants, animal subjects, manual group assignment, or subjective outcome assessment were involved.

Reporting for specific materials, systems and methods

We require information from authors about some types of materials, experimental systems and methods used in many studies. Here, indicate whether each material, system or method listed is relevant to your study. If you are not sure if a list item applies to your research, read the appropriate section before selecting a response.

Materials & experimental systems

n/a	Involved in the study
☒	☐ Antibodies
☒	☐ Eukaryotic cell lines
☒	☐ Palaeontology and archaeology
☒	☐ Animals and other organisms
☒	☐ Clinical data
☒	☐ Dual use research of concern
☒	☐ Plants

Methods

n/a	Involved in the study
☒	☐ ChIP-seq
☒	☐ Flow cytometry
☒	☐ MRI-based neuroimaging

Plants

Seed stocks	Not Applicable.
Novel plant genotypes	Not Applicable.
Authentication	Not Applicable.
