# Supplementary material for: Mol-CADiff: text-conditional molecule generation via causality-aware autoregressive diffusion
Source: Nat Commun. 2026 Jul 29;17:9190. doi: 10.1038/s41467-026-75702-5 (PMC13522512; doi:10.1038/s41467-026-75702-5)
Supplement: Supplementary file 4 [file 41467_2026_75702_MOESM5_ESM.pdf]

nature portfolio

Peer Review File

Mol-CADiff: text-conditional molecule generation via causality-aware autoregressive diffusion

Corresponding Author: Dr Qiang Cheng

This file contains all reviewer reports in order by version, followed by all author rebuttals in order by version.

Version 0:

Reviewer comments:

Reviewer #2

(Remarks to the Author)
This work introduces Mol-CADiff, a diffusion-based model for text-conditional molecule generation that combines autoregressive decomposition with causal attention. The method replaces standard attention with a generalized causal attention mask and adopts an autoregressive token generation strategy to explicitly model dependencies within and across modalities. The results suggest that the method improves controllability and expressiveness in language-driven molecule design. However, several concerns should be addressed by the author:

1. Major Concerns on Models:
(1) Train-inference mismatch: Causal masking is enforced during training but appears absent in inference. This violates the autoregressive inductive bias at test time.
(2) Distribution shift: The decoder is trained on encoder posteriors but not on diffusion-generated latents. At inference it consumes a different latent distribution, which may hurt validity.
(3) Stacked noise: VAE reparameterization adds noise to z while diffusion also corrupts z, weakening signal.
(4) Autoregression over latent dimensions: The dimension-wise AR order is arbitrary and may not align with chemical structure.
(5) Prior–posterior mismatch: VAE pushes z toward a simple prior (), while diffusion learns a complex conditional prior p(z|text). the author should analyze the overlap and discuss how mismatch affects decoding.
2. The paper claims "causal relationships" between text and molecules, but no intervention-based tests and no ablation showing text conditions causally drive specific structural changes.
3. The current evaluation overlooks several critical pharmaceutical-relevant metrics that are essential for assessing the practical utility of generated molecules such as the distribution of QED, SA and logP between generated molecules and reference molecules.
4. The novelty threshold of 0.8 (based on MACCS similarity) appears not strict enough for meaningful differentiation of novel chemical entities.
5. The comparisons should be expanded to include: LLM-based approaches for text-conditional molecule design and SOTA graph generators to evaluate graph-structured generation.
6. The experiments mainly focus on generation statistics and basic property evaluation, but lack practical validation of the model's utility. In particular, no structure-based tasks such as molecular docking are conducted to assess target-specific binding performance.

(Remarks on code availability)

Reviewer #3

(Remarks to the Author)
The authors propose a novel diffusion-based framework that uses causal attention for introducing text into the molecule generation process. The results show similar or better performance to existing methods. The paper is well structured and well written. One major exception is the description of the attention mechanism on page (135-140) which is not well described and hard to understand, as it is only high-level and not sufficiently detailed. This part should be revised and

extended.

Presentation of results
- In table 2 and 3, the best model per metric should be highlighted in bold (currently this is only done for the overall metric)
- In table4, condition molecule is insoluble in water, the MolT5-large results show 3x the same molecule, this seems to be a copy-paste-error.
- Figure 4,6,7: Y-axis labeling is misleading since it does not start at 0. This needs to more clearly indicated, e.g. by a break.
Minor points:
- Abbreviations like AR need to be introduced before first used.
- It is repeatedly highlighted that Mol-CADiff generates valid molecules, however, this seems be because of the choice of (external) graph decoder and therefore should be less highlighted

(Remarks on code availability)

Version 1:

Reviewer comments:

Reviewer #3

(Remarks to the Author)
All my points have been comprehensively addressed in the revision.

(Remarks on code availability)

Open Access This Peer Review File is licensed under a Creative Commons Attribution 4.0 International License, which permits use, sharing, adaptation, distribution and reproduction in any medium or format, as long as you give appropriate credit to the original author(s) and the source, provide a link to the Creative Commons license, and indicate if changes were made.
In cases where reviewers are anonymous, credit should be given to 'Anonymous Referee' and the source.
The images or other third party material in this Peer Review File are included in the article's Creative Commons license, unless indicated otherwise in a credit line to the material. If material is not included in the article's Creative Commons license and your intended use is not permitted by statutory regulation or exceeds the permitted use, you will need to obtain permission directly from the copyright holder.
To view a copy of this license, visit https://creativecommons.org/licenses/by/4.0/

Response Letter for Manuscript:
Mol-CADiff: Causality-Aware Autoregressive Diffusion for Molecule Generation

We sincerely thank the reviewers for their time and effort in providing a thorough evaluation of our manuscript. We greatly appreciate their insightful comments and constructive suggestions, which we have carefully addressed and leveraged to improve our work in the revised submission.

In response to the reviewers' feedback, we conducted several additional experiments and provided detailed, point-by-point replies to all comments. For clarity, each comment is presented in a boxed format, followed by our corresponding response. We also indicate the location of revisions in the revised manuscript and provide excerpts where applicable. Changes in the manuscript are highlighted in green. All relevant figures and tables are included in the updated manuscript. We look forward to any further comments from the reviewers.

Responses to Reviewer Comments

Reviewer 2

> **[Comment-1]** This work introduces Mol-CADiff, a diffusion-based model for text-conditional molecule generation that combines autoregressive decomposition with causal attention. The method replaces standard attention with a generalized causal attention mask and adopts an autoregressive token generation strategy to explicitly model dependencies within and across modalities. The results suggest that the method improves controllability and expressiveness in language-driven molecule design. However, several concerns should be addressed by the author.

Response: We thank the reviewer for the thoughtful summary of our work and for recognizing the potential of Mol-CADiff in improving controllability and expressiveness for language-driven molecule design. We have carefully considered each of the concerns raised in the following points and have revised the manuscript accordingly. Please find our point-by-point responses below.

> **[Comment-2]** Train-inference mismatch: Causal masking is enforced during training but appears absent in inference. This violates the autoregressive inductive bias at test time.

Response: We sincerely thank the reviewer for raising this concern and appreciate the reviewer for encouraging us to improve the clarity of this point. We would like to clarify

that the behavior of our model at inference time is intentional and does not violate the autoregressive inductive bias.

During training, clean latent tokens from earlier autoregressive segments coexist with noised tokens from the current segment. In this setting, a causal attention mask is necessary to prevent information from flowing from future positions into the prediction of the current token.

During inference, however, generation (autoregressive part) proceeds sequentially, and only previously generated latent states are available at any step. Our implementation maintains an incremental key–value (KV) cache during decoding, meaning that future tokens have not yet been produced and therefore do not exist in the cache. As a result, the model is structurally restricted to attending only to past information, and the autoregressive constraint is enforced implicitly by the KV-cache mechanism. In simpler terms, the model cannot 'see' future tokens during inference because they have not been generated yet, which naturally enforces the causal constraint without requiring a mask. In such a setting, an explicit causal mask is not required because the attention mechanism never has access to future positions in the first place.

We have added a clarifying explanation in the manuscript (Subsection Autoregressive Behavior at Inference.) to make this distinction between training-time masking and inference-time autoregressive decoding more transparent.

The relevant excerpt from the revised manuscript is shown below:

> **Autoregressive Behavior at Inference.** During inference, our model generates latent tokens sequentially. At each step, it maintains a key–value (KV) cache that stores only the representations of tokens generated so far. When producing the next token, the attention mechanism retrieves information exclusively from this cache. Because future tokens have not yet been generated and thus do not exist in the cache, the model is inherently restricted to attending only to past information. This structure preserves the autoregressive property without requiring an explicit causal mask at inference time. In contrast, during training, clean latent tokens from earlier autoregressive segments are provided alongside the noised tokens of the current segment, making a causal mask necessary to prevent access to future positions. This separation ensures that the model learns correct dependency patterns during training while supporting efficient decoder-style generation during inference.

> **[Comment-3]** Distribution shift: The decoder is trained on encoder posteriors but not on diffusion-generated latents. At inference it consumes a different latent distribution, which may hurt validity.

Response: We thank the reviewer for highlighting the possibility of a latent distribution shift between encoder-produced latents (used to train the decoder) and diffusion-generated latents (used at inference). To examine this concern empirically, we performed an additional ablation in which the graph decoder was trained jointly with the diffusion model, rather than only during VAE pretraining. The results on the ChEBI-20 dataset show only marginal differences in Similarity, Novelty, and Diversity while maintaining 100% Validity.

This confirms that the denoised latent produced by the diffusion model already lies in the encoder's latent manifold, as expected from the denoising objective. Moreover, the VAE decoder is inherently robust to local perturbations in latent space due to stochastic sampling during training. The consistently 100% chemical validity across all datasets further indicates that diffusion-generated latents maintain structural integrity without inducing distributional discrepancies.

We have added this ablation to the revised manuscript (please see new Subsection "Effect of Joint Decoder Training during Diffusion Learning" and Figure 5 (d)).

The relevant excerpts from the revised manuscript are shown below:

Effect of Joint Decoder Training during Diffusion Learning

To evaluate whether jointly training the graph decoder during the diffusion learning stage improves the alignment between generated latents and the decoder's latent space, we conduct an additional ablation study on the ChEBI-20 dataset. In this variant, the graph decoder is updated simultaneously with the diffusion model, rather than remaining fixed after the initial VAE pretraining stage.

Figure 5d presents the comparative results. Joint decoder training yields equivalent chemical validity (100%) and only marginal fluctuations in similarity, novelty, and diversity. These minor differences do not indicate a systematic improvement in performance. This demonstrates that the diffusion denoiser effectively learns to produce latents well-aligned with the pretrained encoder manifold, and that the VAE decoder is robust to minor variations in the latent distribution. Given the lack of significant benefits and the increased computational overhead, we retain the fixed pretrained decoder as our default configuration.

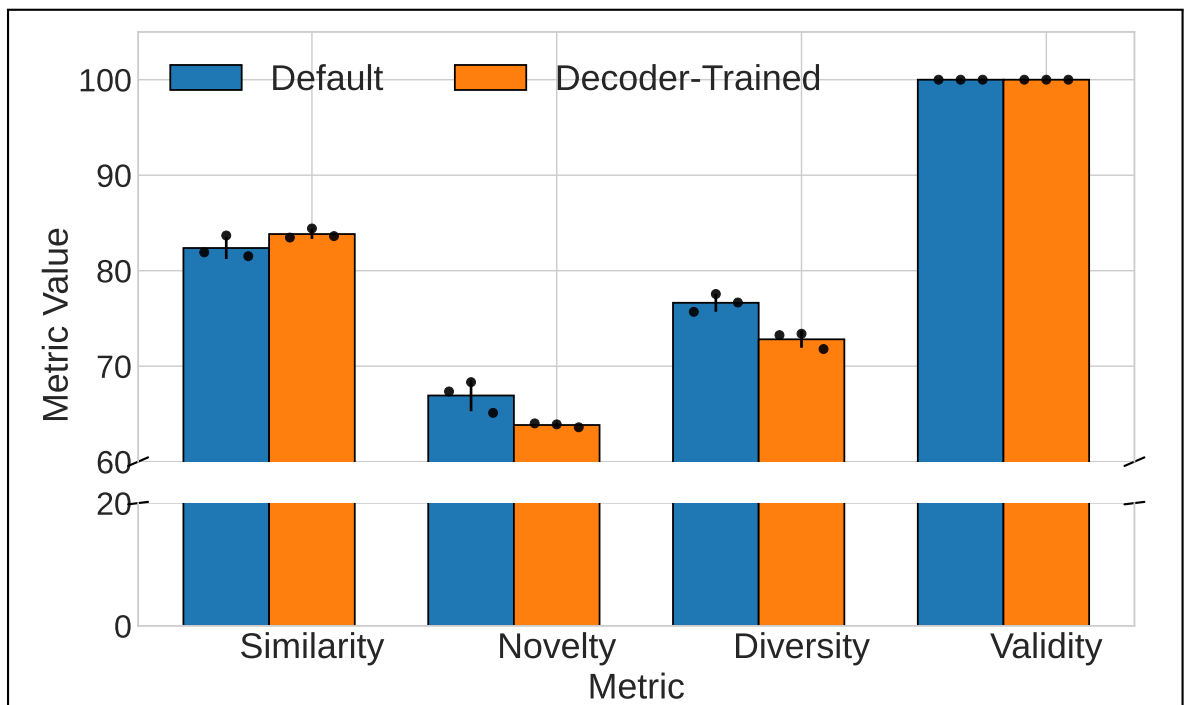

(d) Effect of joint training strategy

[**Comment-4**] Stacked noise: VAE reparameterization adds noise to z while diffusion also corrupts z, weakening signal.

Response: We thank the reviewer for pointing out the potential concern of "stacked noise" from combining VAE reparameterization with diffusion corruptions. We clarify that in our framework these two noise sources do not simply accumulate in an uncontrolled way and do not weaken the signal in practice.

First, the VAE reparameterization noise is used only during the training of the encoder–decoder pair in order to learn a robust latent space. The decoder is trained to reconstruct molecules from samples $z \sim \mathcal{N}(\mu, \sigma^2 I)$ drawn from a narrow posterior around the mean μ. After pretraining, the resulting latent representation L_{g_0} is treated as the "clean" code for diffusion training. In other words, the diffusion model does not see an ever-increasing stack of noise, but instead operates on a fixed latent sample that already defines the clean signal.

Second, the diffusion process introduces a controlled and well calibrated noise schedule $\{\beta_t\}_{t=1}^{T}$, which is explicitly optimized to be inverted by the denoising network. The learning objective encourages the model to recover L_{g_0} from its noised versions, so the net effect of diffusion is to approximate an identity mapping on the clean latent manifold, rather than to further degrade the signal.

Third, during inference, the generation path is given by text condition → diffusion latent → decoder, without any additional stochastic sampling from the VAE posterior. Thus, VAE reparameterization noise is not repeatedly applied at test time.

Empirically, Mol-CADiff maintains 100% chemical validity and strong similarity, novelty, and diversity across all datasets, which would not be the case if the signal in latent space were systematically washed out by stacked noise.

> **[Comment-5]** Autoregression over latent dimensions: The dimension-wise AR order is arbitrary and may not align with chemical structure.

Response: We appreciate the reviewer's insightful comment regarding the potential misalignment between a dimension-wise autoregressive (AR) order and the underlying chemical structure. We agree that arbitrary or randomly permuted orders may not reflect chemically meaningful dependencies.

In our implementation, however, the AR order is not arbitrary. Unlike the original CausalFusion framework [1], which explicitly applies a random shuffle to the latent tokens before causal attention (see CausalFusion's original implementation employing arbitrary ordering in `models.py`, lines 389–393, available at this link, last accessed Dec. 30, 2025), our model does not perform this kind of order shuffling (see our provided implementation in supplementary files at polymers/poly_hgraph/gine_diffusion.py→ CausalDiffusionModel→ forward function). The AR direction is therefore stable and directly inherited from the pretrained VAE latent space. This constitutes a key difference in our implementation of molecule generation compared to the CausalFusion framework, which was primarily designed for images.

Since the VAE learns a smooth, structure-aware latent manifold for molecular graphs, maintaining this coordinate system provides a consistent and chemically informed ordering. In practice, this avoids the potential misalignment introduced by random shuffling while enabling the causal transformer to model dependencies along a semantically coherent axis.

[Comment-6] Prior–posterior mismatch: VAE pushes z toward a simple prior (), while diffusion learns a complex conditional prior $p(z|text)$. the author should analyze the overlap and discuss how mismatch affects decoding.

Response: We thank the reviewer for raising this important point. We agree that, in principle, a mismatch may arise because the VAE latent space is trained under a simple isotropic Gaussian prior $p(z)$, whereas the diffusion model learns a richer conditional prior $p(z \mid \text{text})$. We clarify below why this does not introduce instability in decoding in our setting.

First, the VAE prior is used only during encoder–decoder pretraining to shape a smooth and well-behaved latent manifold. After pretraining, the decoder is exposed to latent samples that remain close to the posterior distribution $q(z \mid x)$ encountered during training. The diffusion model operates entirely within this same manifold: it starts from a noise distribution and iteratively denoises toward the region of latent space corresponding to valid molecular structures. In other words, the diffusion model learns a conditional distribution that *maps back into* the geometry defined by the VAE, rather than exploring regions unsupported by the decoder.

Second, although $p(z)$ and $p(z \mid \text{text})$ differ in complexity, their support substantially overlaps. The decoder is robust to small variations around the posterior due to the VAE training objective, which explicitly teaches the decoder to reconstruct from a neighborhood of $q(z \mid x)$ rather than a single point estimate. As a result, samples produced by the diffusion model fall within the decoder's validity region and do not suffer from instability due to prior–posterior mismatch.

Third, our empirical results strongly indicate that any remaining mismatch does not harm decoding: validity reaches 100% across datasets, and similarity, novelty, and diversity remain high. This would not hold if the diffusion model frequently produced latent codes outside the decoder's learned manifold.

[Comment-7] The paper claims "causal relationships" between text and molecules, but no intervention-based tests and no ablation showing text conditions causally drive specific structural changes.

Response: We thank the reviewer for this thoughtful and important comment. While the paper included case studies in the appendix (please see S-Table 1 vs S-Table 2 in the previous submission, which are now Supplementary Figure 3 and Supplementary Figure 4 in the revised manuscript, respectively.), following the reviewer's helpful suggestion, we now provide a dedicated intervention-based ablation experiment in the revised manuscript.

Specifically, we contrast pairs of opposing prompts—such as "The molecule is like a drug" vs. "The molecule is not like a drug" and "The molecule is insoluble in water" vs. "The molecule is soluble in water", and measure their effects on chemically meaningful properties (QED for drug-likeness and logP for solubility). The results, now shown in Figure 5 (e) of the revision, reveal clear and consistent differences. The "like a drug" prompt produces molecules with markedly higher QED values, whereas the opposite prompt yields structures with substantially lower drug-likeness. Similarly, the "insoluble" prompt leads to molecules with higher logP (more hydrophobic), while the "soluble" prompt results in lower logP (more hydrophilic) structures. These controlled contrasts demonstrate that text conditions do indeed steer the generated molecules toward distinct regions of chemical property space.

The relevant excerpts from the revised manuscript are shown below:

Intervention-Based Ablation

To examine whether text conditions meaningfully influence the molecular properties produced by Mol-CADiff, we perform a controlled intervention-based ablation using contrasting prompts. For drug-likeness, we compare the top 50 generated molecules conditioned on "The molecule is like a drug" versus "The molecule is not like a drug." We quantify the results using the QED score, where higher values indicate structures with greater drug-like relevance. As shown in Figure 5e, conditioning on the positive drug-like prompt produces molecules with substantially higher QED scores, while the negative prompt shifts the distribution toward significantly lower values, confirming that the text effectively modulates the generative trajectory toward or away from drug-like chemical space.

Similarly, for solubility, we contrast the prompts "The molecule is insoluble in water" and "The molecule is soluble in water," using logP as the evaluative metric (where higher logP indicates increased hydrophobicity). The "insoluble" prompt yields molecules with significantly higher logP, whereas the "soluble" prompt produces molecules with much lower logP values. Taken together, these intervention-based tests indicate that textual conditions exert a systematic and property-congruent influence on the generated structures. These observed trends, including higher QED for drug-like prompts and higher logP for insolubility prompts, align with established chemical principles. These results further validate the effectiveness of text-guided generation within the Mol-CADiff framework.

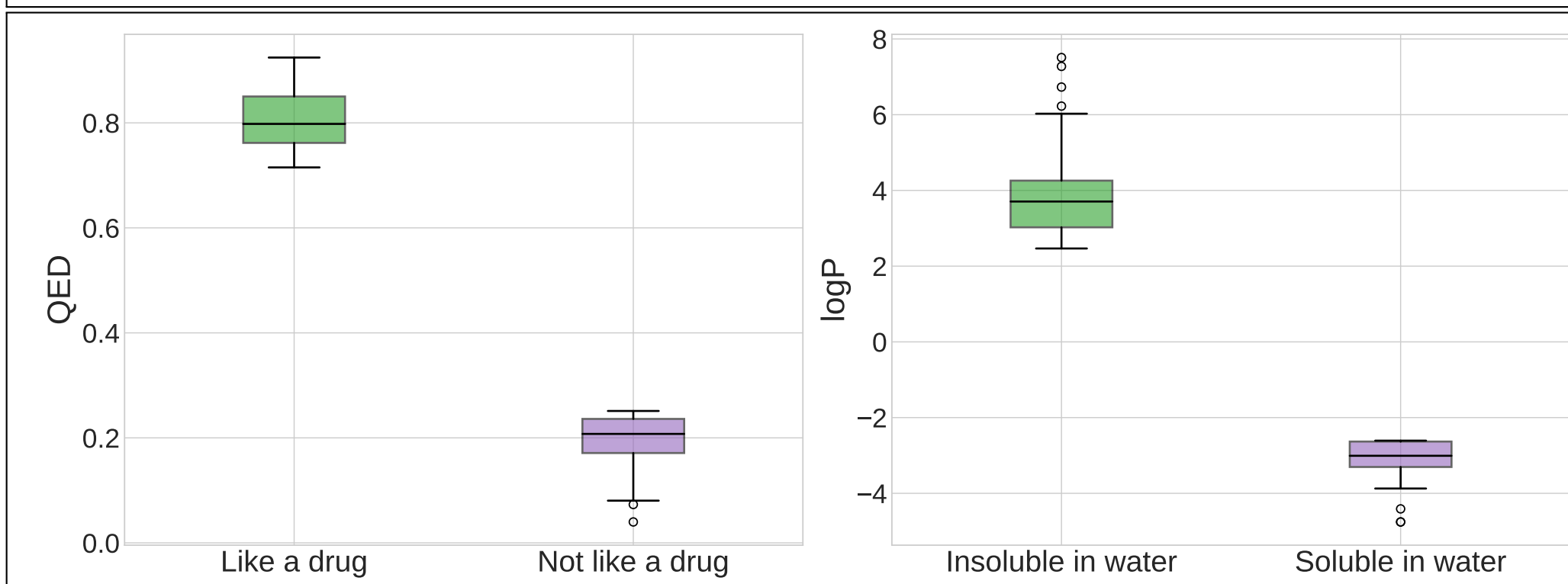

(e) Property intervention analysis

> **[Comment-8]** The current evaluation overlooks several critical pharmaceutical-relevant metrics that are essential for assessing the practical utility of generated molecules such as the distribution of QED, SA and logP between generated molecules and reference molecules.

Response: We sincerely thank the reviewer for this valuable suggestion. We agree that analyzing pharmaceutical-relevant metrics such as QED, SA, and logP is crucial to demonstrate the practical utility of generated molecules. Following this recommendation, we have added a new part in the Result Section (Pharmaceutical-Relevant Property Distributions), where we report the distributions of these properties for our generated molecules compared to the reference molecules. The results, illustrated in Figure 4 (a-c), show that the generated distributions closely track those of the reference set. In particular, the overlap for SA is nearly perfect, underscoring the synthetic feasibility of the generated compounds, while QED and logP distributions are also well aligned with the reference, indicating drug-likeness and appropriate lipophilicity profiles. These findings confirm that Mol-CADiff not only achieves validity, novelty, and diversity but also produces molecules consistent with real-world pharmaceutical properties, thereby strengthening its practical applicability in drug discovery.

The relevant excerpts from the revised manuscript are shown below:

Pharmaceutically Relevant Property Distributions

To further validate the practical utility of the molecules generated by Mol-CADiff, we examine the distributions of three widely used pharmaceutically relevant properties: quantitative estimate of drug-likeness (QED), synthetic accessibility (SA, rescaled to [0,1] where 1 represents maximum accessibility), and lipophilicity (logP). For each generated molecule and its reference counterpart, we computed these properties using RDKit and compared their empirical distributions. Figure 4 shows kernel density estimates of the distributions. In each plot, the *x*-axis represents the property value, while the *y*-axis represents the estimated probability density. The shaded regions correspond to the probability mass. Greater overlap between the shaded curves indicates closer alignment with the reference distribution.

The results demonstrate that our method closely matches the reference distributions across all three properties. For QED (Figure 4a), our model produces distributions that are well aligned with the reference, ensuring drug-like relevance. For SA (Figure 4b), the overlap is nearly perfect, highlighting the model's ability to generate molecules that are synthetically feasible. Similarly, for logP (Figure 4c), the generated molecules follow the overall shape of the reference distribution, capturing the lipophilicity profile critical for bioavailability. Overall, these results confirm that beyond achieving high novelty and diversity, Mol-CADiff generates molecules whose pharmaceutical property distributions remain consistent with real-world compounds, strengthening the case for its practical applicability in drug discovery.

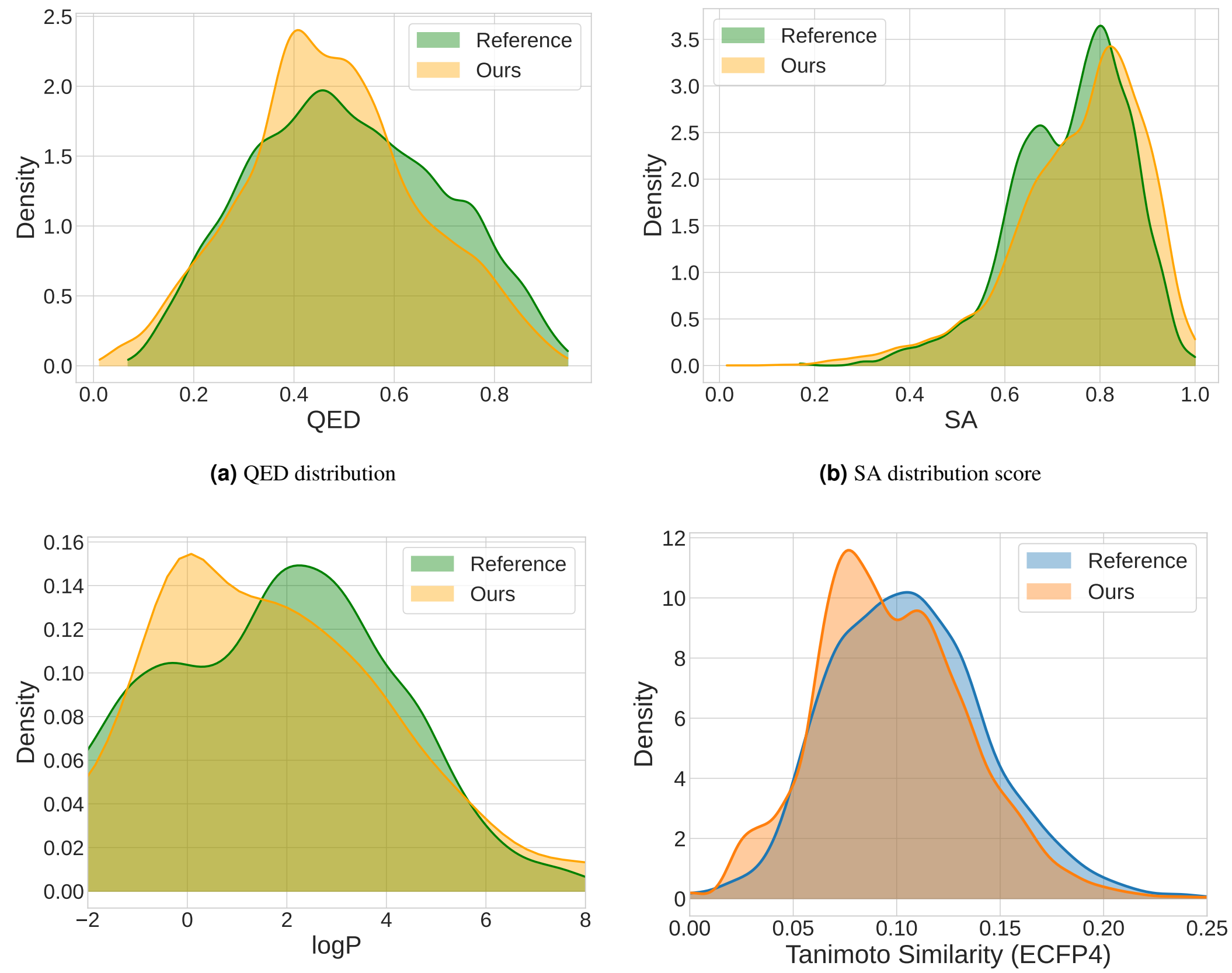

(a) QED distribution

(b) SA distribution score

(c) logP distribution

(d) Distribution of Tanimoto similarity to EGFR inhibitors

Figure 4. **Distributions of pharmaceutically relevant properties and target structural similarity. a–c,** Probability density estimates of molecular property values for molecules generated by Mol-CADiff versus reference molecules for **a,** QED, **b,** SA score, and **c,** logP. Greater distribution overlap indicates closer alignment with the reference chemical space. **d,** Comparison of Tanimoto similarity (ECFP4) distributions between ground-truth reference molecules and those generated by Mol-CADiff, evaluated against a set of known EGFR inhibitors. The high degree of overlap indicates that the model successfully preserves the target-relevant structural profiles. Source data are provided as a Source Data file.

[Comment-9] The novelty threshold of 0.8 (based on MACCS similarity) appears not strict enough for meaningful differentiation of novel chemical entities.

Response: We thank the reviewer for raising this concern. To assess the sensitivity of the novelty metric to the choice of MACCS-based similarity threshold, we have added an ablation experiment in the revised manuscript. We compute novelty at thresholds ranging from 0.70 to 0.90. The results show a smooth and monotonic increase in novelty. We choose 0.8 for a fair comparison similar to the baselines [2]. While increasing the threshold, quantitatively, our method demonstrates even more novelty. Please see Figure 5 (f) and

Subsection “Ablation on Novelty Threshold Using MACCS Similarity”.

The relevant excerpts from the revised manuscript are shown below:

Ablation on Novelty Threshold using MACCS Similarity

We conduct an ablation study to assess the sensitivity of our novelty metrics across MACCS-based similarity cutoffs of 0.70, 0.75, 0.80, 0.85, and 0.90. Figure 5f reports the percentage of generated molecules classified as novel at each threshold. As expected, the novelty rate increases monotonically as the cutoff becomes more relaxed (as implied by the novelty definition). Specifically, the novelty percentage rises from 48.04% at a cutoff of 0.70 to 82.67% at a cutoff of 0.90. Notably, at the standard 0.80 cutoff frequently adopted in prior literature, Mol-CADiff achieves 67.35% novelty. This confirms that the model's capacity for generating novel structures is significant and consistent under standard evaluation settings.

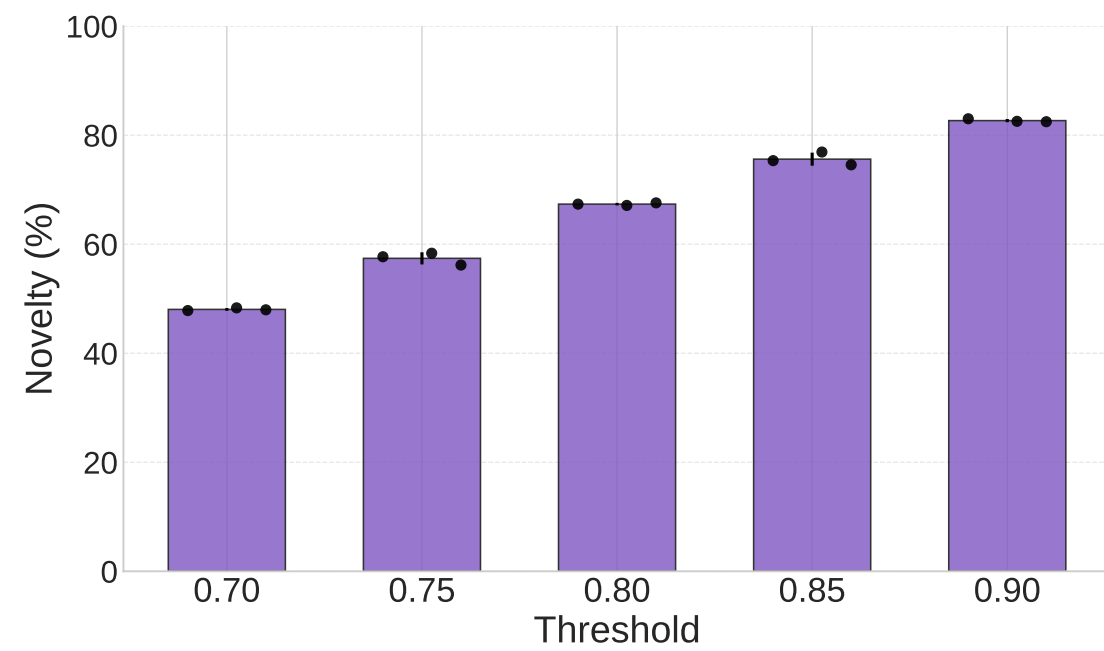

(f) Novelty across thresholds

[Comment-10] The comparisons should be expanded to include: LLM-based approaches for text-conditional molecule design and SOTA graph generators to evaluate graph-structured generation.

Response: We sincerely appreciate this constructive suggestion. We agree that expanding the baseline comparisons provides a more comprehensive evaluation of Mol-CADiff's performance relative to the rapid advancements in the field.

In our previous submission, we compared our method against foundational LLM-based approaches (e.g., MolT5, ChemT5) and established graph generators (e.g., HierVAE). Following the reviewer's recommendation, we have now significantly expanded our comparative analysis to include the latest state-of-the-art (SOTA) methods in both categories:

- **LLM-based Approaches:** We have added **Atomas** (ICLR 2025) [3], a unified framework utilizing hierarchical adaptive alignment, and **MolReGPT** (IEEE TKDE

2024) [4], which leverages general-purpose LLMs for molecule discovery. Since MolReGPT reports results exclusively on the ChEBI-20 dataset, we present our comparison on this benchmark to ensure a fair evaluation.

- **SOTA Graph Generators:** We have included **DiGress** (ICLR 2023) [5], a discrete denoising diffusion model, and **GruM** (ICML 2024) [6], a graph generation model based on diffusion mixtures.

We have updated the **Introduction** to discuss these advancements and included their performance metrics in our experimental results (refer to **Table 1** for text-conditional generation and **Table 2** for unconditional graph generation). These additions demonstrate that Mol-CADiff remains highly competitive and frequently better in overall performance against SOTA methods.

The relevant excerpts from the revised manuscript are shown below:

Table 1. Quantitative performance on conditional molecule generation. Comparative analysis across four benchmark datasets. Metrics include similarity (Sim.), novelty (Nov.), diversity (Div.), and validity (Val.), with Overall (Ove.) representing their arithmetic mean. All results are percentages (higher is better). Mol-CADiff outperforms SOTA methods in novelty, diversity, and validity while maintaining competitive similarity, achieving the highest Overall scores across all datasets.

	ChEBI-20					PubChem					PCDes					MoMu				
Methods	Sim.	Nov.	Div.	Val.	Ove.	Sim.	Nov.	Div.	Val.	Ove.	Sim.	Nov.	Div.	Val.	Ove.	Sim.	Nov.	Div.	Val.	Ove.
MolT5-small	73.32	31.43	17.22	78.27	50.06	68.36	20.63	9.32	78.86	44.29	64.84	24.91	9.67	73.96	43.35	16.64	97.49	29.95	60.19	51.07
MolT5-base	80.75	32.83	17.66	84.63	53.97	73.85	21.86	9.89	79.88	46.37	71.71	25.85	10.50	81.92	47.50	19.76	97.78	29.98	68.84	54.09
MolT5-large	96.88	12.92	11.20	98.06	54.77	**91.57**	20.85	9.84	95.18	54.36	**88.37**	20.15	9.49	96.48	53.62	**25.07**	97.47	30.33	90.40	60.82
Atomas	**96.98**	41.52	26.98	98.03	65.88	90.33	44.39	30.11	94.88	64.93	85.88	34.44	20.12	97.89	59.58	23.05	97.88	34.01	89.88	61.21
ChemT5-small	96.22	13.94	13.50	96.74	55.10	89.32	20.89	13.10	93.47	54.19	86.27	23.28	13.17	93.73	54.11	23.25	96.97	30.04	88.45	59.68
ChemT5-base	95.48	15.12	13.91	97.15	55.42	89.42	22.40	13.98	92.43	54.56	85.01	25.55	14.08	92.93	54.39	23.40	97.65	30.07	87.61	59.68
Mol-Instruction	65.75	32.01	26.50	77.91	50.54	23.40	37.37	27.97	71.10	39.96	60.86	35.60	24.57	79.19	50.06	14.89	97.52	30.17	68.32	52.73
3M-Diffusion	87.09	55.36	34.03	100.0	69.12	87.05	64.41	33.44	100.0	71.22	81.57	63.66	32.39	100.0	69.41	24.62	98.16	37.65	100.0	65.11
**Mol-CADiff**	81.92	**67.35**	**75.69**	**100.0**	**81.24**	78.35	**71.97**	**75.39**	**100.0**	**81.43**	71.41	**74.77**	**75.45**	**100.0**	**80.41**	24.41	**98.34**	**83.30**	**100.0**	**76.51**

Table 2. Quantitative performance on unconditional generation. Comparison across ChEBI-20 and PubChem datasets. Metrics include Uniqueness (Uni.), Novelty (Nov.), transformed KL Divergence (KL), transformed Fréchet ChemNet Distance (FCD), and Validity (Val.). Ove. denotes the arithmetic mean. All results are percentages (higher is better). Mol-CADiff achieves the highest Overall performance, demonstrating a strong balance between validity and distributional alignment.

	**ChEBI-20**						**PubChem**					
Methods	Uni.	Nov.	KL	FCD	Val.	Ove.	Uni.	Nov.	KL	FCD	Val.	Ove.
CharRNN	72.46	11.57	95.21	75.95	98.21	70.68	63.28	23.47	90.72	**76.02**	94.09	69.52
VAE	57.57	47.88	95.47	74.19	63.84	67.79	44.45	42.47	91.67	55.56	94.10	65.65
AAE	1.23	1.23	38.47	0.06	1.35	8.47	2.94	3.21	39.33	0.08	1.97	9.51
LatentGAN	66.93	57.52	94.38	76.65	73.02	73.70	52.00	50.36	91.38	57.38	53.62	60.95
BwR	22.09	21.97	50.59	0.26	22.66	23.51	82.35	82.34	45.53	0.11	87.73	59.61
HierVAE	82.17	72.83	93.39	64.32	100.0	82.54	75.33	72.44	89.05	50.04	100.0	77.37
PS-VAE	76.09	74.55	83.16	32.44	100.0	73.25	66.97	66.52	83.41	14.41	100.0	66.26
GruM	83.10	75.20	94.85	74.15	99.40	85.34	84.90	79.20	94.60	67.85	99.50	85.21
DiGress	82.40	74.50	94.10	72.60	98.70	84.46	83.85	78.95	93.90	66.95	98.90	84.51
3M-Diffusion	83.04	70.80	**96.29**	**77.83**	100.0	85.59	85.42	81.20	92.67	58.27	100.0	83.51
**Mol-CADiff**	**85.90**	**79.11**	94.69	74.45	**100.0**	**86.83**	**87.79**	**83.23**	**95.19**	71.65	**100.0**	**87.57**

Comparison with GPT-4-based MolReGPT

We additionally compare Mol-CADiff with another LLM-based approach, MolReGPT[37], instantiated with GPT-4 (version 0413) on the ChEBI-20 dataset. We evaluate the same four conditional generation metrics used in our main analysis: similarity, novelty, diversity, and validity. The results are summarized in Figure 5g. We observe that MolReGPT achieves similarity scores comparable to MolT5-large and Atomas, reflecting a tendency to generate structures that remain very close to the ground-truth molecules. However, this high similarity comes at the cost of substantially reduced novelty and diversity, indicating that GPT-4 primarily explores regions of chemical space already well-covered by the training data.

In contrast, Mol-CADiff attains markedly higher novelty and diversity while maintaining perfect validity and competitive similarity. This trade-off aligns with our core design goal: Mol-CADiff is engineered to explore chemically plausible yet novel molecules rather than simply reproducing near-duplicates of the reference set. Since MolReGPT reports results exclusively on the ChEBI-20 dataset, we present our comparison on this benchmark to ensure a fair evaluation; the observed trends are consistent with the multi-dataset comparisons detailed in our main results.

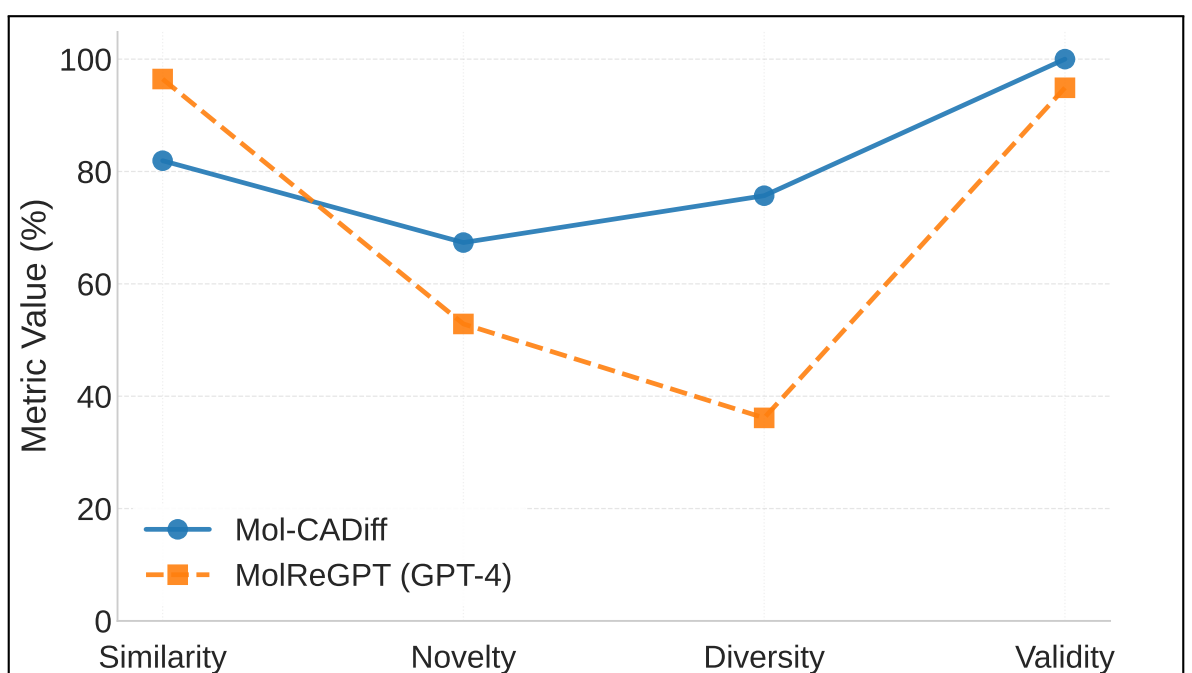

(g) Comparison with MolReGPT

[Comment-11] The experiments mainly focus on generation statistics and basic property evaluation, but lack practical validation of the model's utility. In particular, no structure-based tasks such as molecular docking are conducted to assess target-specific binding performance.

Response: We appreciate the reviewer's emphasis on structure-based validation. We carefully considered performing molecular docking; however, we opted for ligand-based validation (ECFP4 fingerprint similarity) for a specific methodological reason rooted in the nature of our model.

Mol-CADiff is a 2D molecular graph generator, not a 3D conformer generator. To perform docking, we would need to pass our graphs through an external 3D embedding tool (e.g., RDKit's ETKDG or Omega). Consequently, any docking score would conflate the performance of our generative model with the accuracy of the external conformer generator. If the external tool fails to find the bioactive conformer, it would unfairly penalize our generative model.

Therefore, to assess target relevance without this confounding variable, we utilized ligand-based target prediction (ECFP4 similarity against approved EGFR inhibitors). This is a standard industry metric for 2D generative models that quantifies whether the generated chemical features align with the pharmacophore requirements of the target. As shown in Figure 4(d), our method achieves a distribution that closely matches ground truth inhibitors, demonstrating that Mol-CADiff successfully captures target-specific chemical space.

The relevant excerpts from the revised manuscript are shown below:

Ligand-Based Target-Relevant Validation

To complement the evaluation of chemical validity and drug-like properties, we conducted a ligand-based validation to assess whether Mol-CADiff generates molecules with target-relevant potential. As a representative case study, we considered the epidermal growth factor receptor (EGFR), a well-studied oncology target with multiple approved inhibitors. We computed ECFP4 (Extended-Connectivity Fingerprint, diameter 4) similarities, which are Tanimoto similarities calculated using ECFP4 fingerprints, to a set of known EGFR inhibitors for both: (i) ground-truth molecules from the ChEBI-20 dataset and (ii) molecules generated by Mol-CADiff. This was done to evaluate whether the model preserves target-specific motifs.

Figure 4d shows that the similarity distributions of generated and ground-truth molecules to EGFR inhibitors almost completely overlap, indicating that our model reproduces the same target-relevant chemical patterns present in the reference dataset. This further confirms that Mol-CADiff is not generating molecules arbitrarily but instead captures pharmacologically meaningful regions of chemical space.

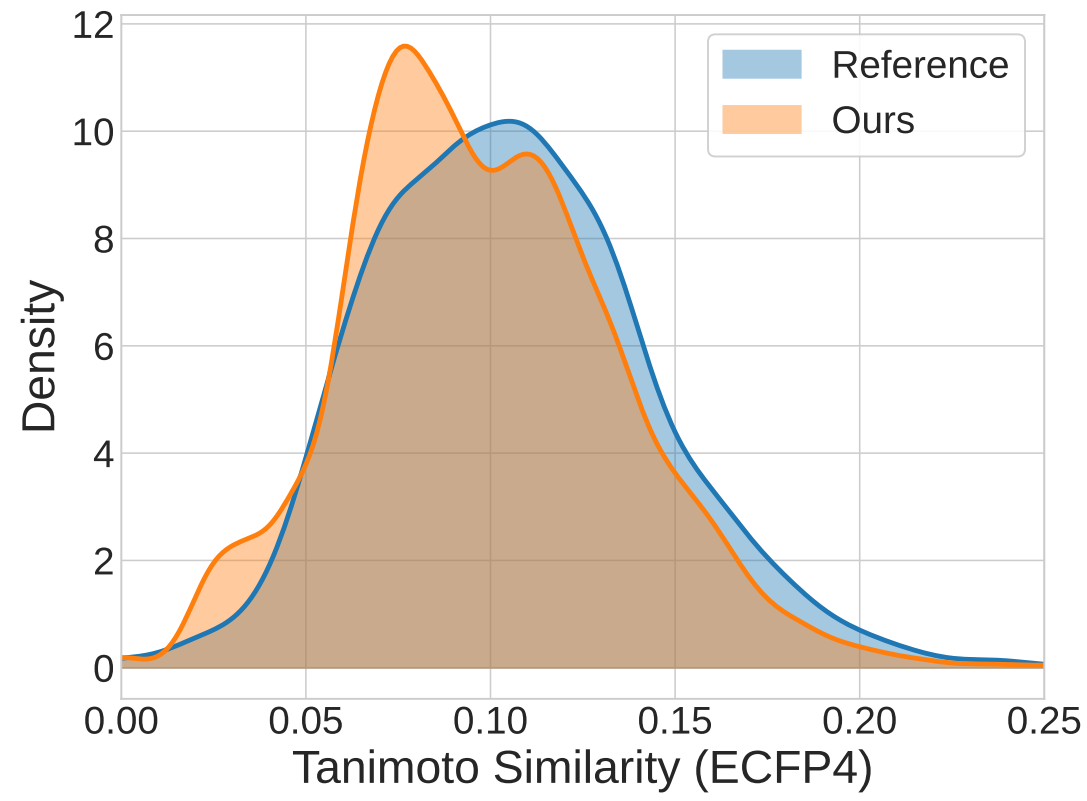

(d) Distribution of Tanimoto similarity to EGFR inhibitors

Reviewer 3

[**Comment-1**] The authors propose a novel diffusion-based framework that uses causal attention for introducing text into the molecule generation process. The results show similar or better performance to existing methods. The paper is well structured and well written. One major exception is the description of the attention mechanism on page (135-140) which is not well described and hard to understand, as it is only high-level and not sufficiently detailed. This part should be revised and extended.

Response: We sincerely thank the reviewer for the positive evaluation of the overall structure and clarity of our paper. We also appreciate the constructive feedback regarding the description of the attention mechanism. Following your suggestion, we have provided a detailed and clarified description of the attention mechanism, including a precise explanation of the causal masking and its role in enforcing autoregressive structure. This expanded explanation has been added to the Methods section to improve clarity and readability. Please see our revised part in "Integrating Autoregression with Diffusion" under the Methods section.

The relevant excerpt from the revised manuscript is shown below:

$$q(L_{g_{0:T}}) = q(L_{g_0}) \prod_{t=1}^{T} q(L_{g_t} | L_{g_{t-1}}). \quad (4)$$

This formulation effectively models the progressive noise injection process while preserving structural dependencies essential for molecular generation. While diffusion models are well suited for image generation and denoising tasks, they inherently lack the ability to capture dependencies in a causal autoregressive (AR) manner.

To address this, we replace standard attention with a causal attention mechanism and adopt an autoregressive token generation strategy, which enables diffusion and autoregression to operate jointly. Formally, let $X = [L_c, L_{g_{0,\kappa_{1:s-1}}}, L_{g_{t,\kappa_s}}]$ denote the concatenated sequence at AR step s, consisting of conditional text tokens L_c, the clean latent tokens from previous AR steps $L_{g_{0,\kappa_{1:s-1}}}$, and the noisy tokens for the current AR step $L_{g_{t,\kappa_s}}$. Following standard attention[71], we obtain query, key, and value matrices through learned linear projections,

$$Q = XW_Q, \qquad K = XW_K, \qquad V = XW_V.$$

A standard attention layer computes

$$\mathrm{Attn}(Q,K,V) = \mathrm{softmax}\left(\frac{QK^\top}{\sqrt{d}}\right)V,$$

but this allows every token to attend to every other token. To enforce correct autoregressive dependencies, we introduce a binary causal mask $M \in \{0,1\}^{N\times N}$ that restricts information flow, where N denotes the total number of tokens in the sequence X. The masked attention becomes

$$\mathrm{Attn}(Q,K,V;M) = \mathrm{softmax}\left(\frac{QK^\top}{\sqrt{d}} + A\right)V,$$

where

$$A_{ij} = -\infty \ \text{ if } M_{ij} = 1, \qquad A_{ij} = 0 \ \text{ otherwise},$$

ensuring that masked positions receive zero probability after the softmax. The mask is constructed so that: (i) noisy tokens at AR step s cannot access future latent tokens, (ii) clean tokens from earlier AR steps may guide the denoising of the current step, and (iii) conditional text tokens L_c remain visible to all positions. This prevents information leakage from future diffusion or AR steps and allows conditional tokens to act as global context while previously denoised tokens steer the current denoising process.

[Comment-2] In table 2 and 3, the best model per metric should be highlighted in bold (currently this is only done for the overall metric)

Response: We thank the reviewer for this suggestion. We have revised Tables 2 and 3 following your suggestion. The revised tables are now Tables 1 and 2 in the revised manuscript.

The relevant excerpts from the revised manuscript are shown below:

Table 1. Quantitative performance on conditional molecule generation. Comparative analysis across four benchmark datasets. Metrics include similarity (Sim.), novelty (Nov.), diversity (Div.), and validity (Val.), with Overall (Ove.) representing their arithmetic mean. All results are percentages (higher is better). Mol-CADiff outperforms SOTA methods in novelty, diversity, and validity while maintaining competitive similarity, achieving the highest Overall scores across all datasets.

	ChEBI-20					PubChem					PCDes					MoMu				
Methods	Sim.	Nov.	Div.	Val.	Ove.	Sim.	Nov.	Div.	Val.	Ove.	Sim.	Nov.	Div.	Val.	Ove.	Sim.	Nov.	Div.	Val.	Ove.
MolT5-small	73.32	31.43	17.22	78.27	50.06	68.36	20.63	9.32	78.86	44.29	64.84	24.91	9.67	73.96	43.35	16.64	97.49	29.95	60.19	51.07
MolT5-base	80.75	32.83	17.66	84.63	53.97	73.85	21.86	9.89	79.88	46.37	71.71	25.85	10.50	81.92	47.50	19.76	97.78	29.98	68.84	54.09
MolT5-large	96.88	12.92	11.20	98.06	54.77	**91.57**	20.85	9.84	95.18	54.36	**88.37**	20.15	9.49	96.48	53.62	**25.07**	97.47	30.33	90.40	60.82
Atomas	**96.98**	41.52	26.98	98.03	65.88	90.33	44.39	30.11	94.88	64.93	85.88	34.44	20.12	97.89	59.58	23.05	97.88	34.01	89.88	61.21
ChemT5-small	96.22	13.94	13.50	96.74	55.10	89.32	20.89	13.10	93.47	54.19	86.27	23.28	13.17	93.73	54.11	23.25	96.97	30.04	88.45	59.68
ChemT5-base	95.48	15.12	13.91	97.15	55.42	89.42	22.40	13.98	92.43	54.56	85.01	25.55	14.08	92.93	54.39	23.40	97.65	30.07	87.61	59.68
Mol-Instruction	65.75	32.01	26.50	77.91	50.54	23.40	37.37	27.97	71.10	39.96	60.86	35.60	24.57	79.19	50.06	14.89	97.52	30.17	68.32	52.73
3M-Diffusion	87.09	55.36	34.03	100.0	69.12	87.05	64.41	33.44	100.0	71.22	81.57	63.66	32.39	100.0	69.41	24.62	98.16	37.65	100.0	65.11
**Mol-CADiff**	81.92	**67.35**	**75.69**	**100.0**	**81.24**	78.35	**71.97**	**75.39**	**100.0**	**81.43**	71.41	**74.77**	**75.45**	**100.0**	**80.41**	24.41	**98.34**	**83.30**	**100.0**	**76.51**

Table 2. Quantitative performance on unconditional generation. Comparison across ChEBI-20 and PubChem datasets. Metrics include Uniqueness (Uni.), Novelty (Nov.), transformed KL Divergence (KL), transformed Fréchet ChemNet Distance (FCD), and Validity (Val.). Ove. denotes the arithmetic mean. All results are percentages (higher is better). Mol-CADiff achieves the highest Overall performance, demonstrating a strong balance between validity and distributional alignment.

	ChEBI-20						PubChem					
Methods	Uni.	Nov.	KL	FCD	Val.	Ove.	Uni.	Nov.	KL	FCD	Val.	Ove.
CharRNN	72.46	11.57	95.21	75.95	98.21	70.68	63.28	23.47	90.72	**76.02**	94.09	69.52
VAE	57.57	47.88	95.47	74.19	63.84	67.79	44.45	42.47	91.67	55.56	94.10	65.65
AAE	1.23	1.23	38.47	0.06	1.35	8.47	2.94	3.21	39.33	0.08	1.97	9.51
LatentGAN	66.93	57.52	94.38	76.65	73.02	73.70	52.00	50.36	91.38	57.38	53.62	60.95
BwR	22.09	21.97	50.59	0.26	22.66	23.51	82.35	82.34	45.53	0.11	87.73	59.61
HierVAE	82.17	72.83	93.39	64.32	100.0	82.54	75.33	72.44	89.05	50.04	100.0	77.37
PS-VAE	76.09	74.55	83.16	32.44	100.0	73.25	66.97	66.52	83.41	14.41	100.0	66.26
GruM	83.10	75.20	94.85	74.15	99.40	85.34	84.90	79.20	94.60	67.85	99.50	85.21
DiGress	82.40	74.50	94.10	72.60	98.70	84.46	83.85	78.95	93.90	66.95	98.90	84.51
3M-Diffusion	83.04	70.80	**96.29**	**77.83**	100.0	85.59	85.42	81.20	92.67	58.27	100.0	83.51
**Mol-CADiff**	**85.90**	**79.11**	94.69	74.45	**100.0**	**86.83**	**87.79**	**83.23**	**95.19**	71.65	**100.0**	**87.57**

[Comment-3] In table 4, condition molecule is insoluble in water, the MolT5-large results show 3x the same molecule, this seems to be a copy-paste-error.

Response: We sincerely thank the reviewer for the careful reading of Table 4 of our prior submission (Now Figure 3 in the revised manuscript). We would like to clarify that the repeated outputs produced by MolT5-large are not the result of a copy–paste oversight. Rather, they accurately reflect the behavior we observed: when given the same textual condition, MolT5-large frequently generates nearly identical molecules across multiple sampling attempts. This phenomenon is also consistent with its lower diversity scores reported in Table 1 in the revised manuscript and aligns with observations from prior work, such as 3M-Diffusion [2] (please see their Figure 2). To ensure fairness and transparency,

we reported the baseline results exactly as produced by the model. We have added clarifying sentences in our revised manuscript.

The relevant excerpt from the revised manuscript is shown below:

> **Qualitative Comparison**
>
> We present qualitative results demonstrating the efficacy of our method in Figure 2, where we visualize five randomly sampled molecules generated by Mol-CADiff alongside their corresponding ground truth structures. It is evident that the generated molecules closely resemble the ground truth, faithfully capturing the textual descriptions provided. This visual similarity underscores our method's ability to effectively leverage textual conditions that reflect the desired properties. Furthermore, we conduct detailed case studies in Figure 3, comparing Mol-CADiff with two best-performing state-of-the-art methods, namely 3M-Diffusion, and MolT5-large, under specific textual instructions. As illustrated, Mol-CADiff consistently outperforms both baselines by generating molecules that better satisfy the given custom conditions.
>
> For instance, when instructed to generate molecules insoluble in water, Mol-CADiff produces molecules with higher logP values, clearly surpassing the baselines in property-steering accuracy. MolT5-large frequently generates near-identical molecules across multiple sampling attempts; this lack of variance is consistent with its lower diversity scores reported in Table 1 and corroborates observations from prior work, such as 3M-Diffusion[44]. Similarly, when generating molecules belonging to the flavone class, our method achieves higher structural similarity to the reference molecules compared to competing methods. These qualitative analyses further validate Mol-CADiff's superiority in generating high-quality, conditionally accurate molecules.
>
> Additional results, including more generated molecules and extended case studies, are provided in the supplementary figures.

[Comment-4] Figure 4,6,7: Y-axis labeling is misleading since it does not start at 0. This needs to more clearly indicated, e.g. by a break.

Response: Thank you for this helpful suggestion. We have updated Figures 4, 6, and 7 of our previous submission by introducing a clear break in the y-axis to avoid any misleading interpretation caused by the non-zero starting point. These figures now explicitly indicate the break using diagonal markers. We have also added error bars following journal requirements for bar charts, and we have combined several figures in an ablation study following the display item limits of the journal requirement. The revised figures are Figure 5 (a-c) in the revised manuscript.

The relevant excerpt from the revised manuscript is shown below:

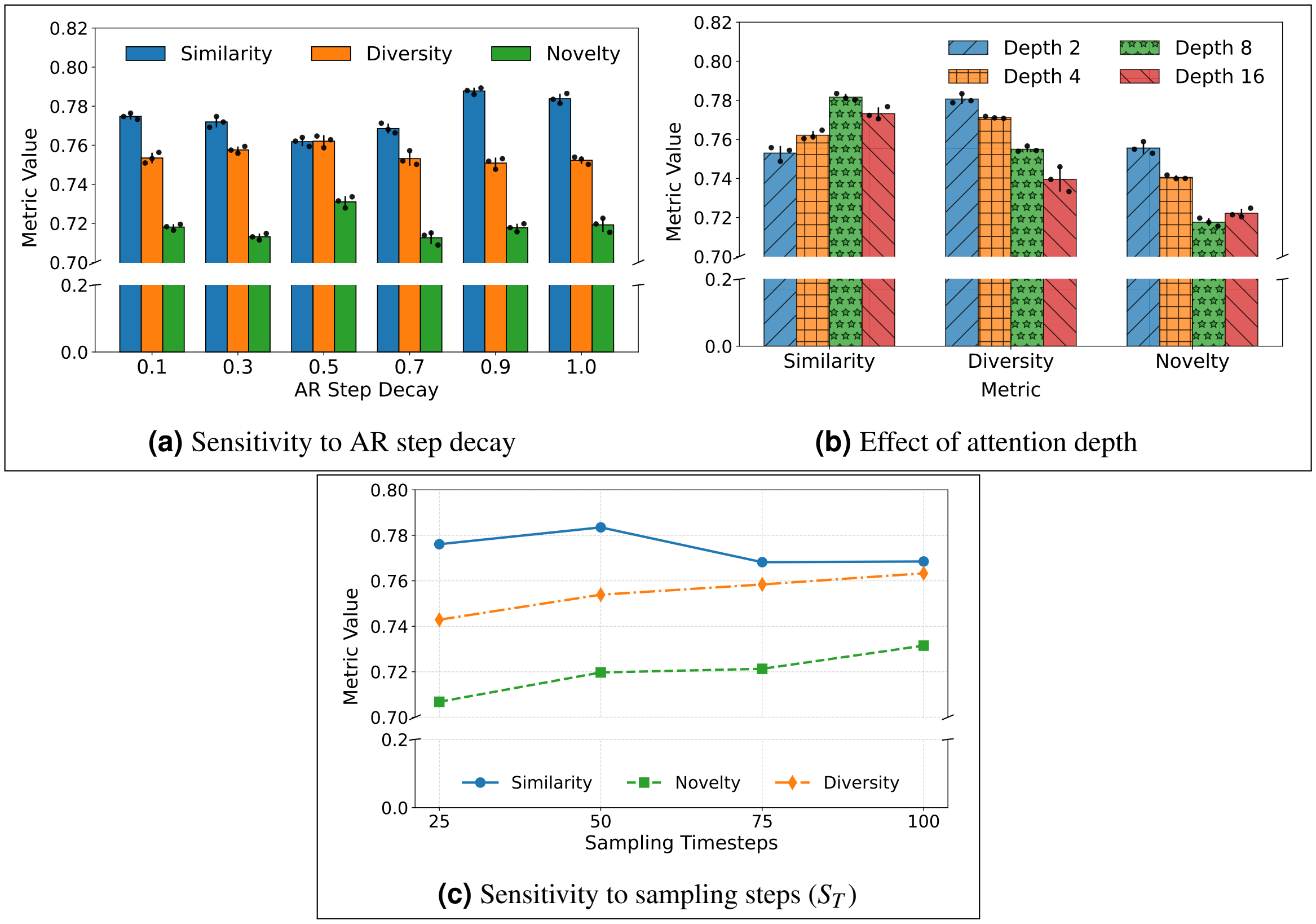

(a) Sensitivity to AR step decay

(b) Effect of attention depth

(c) Sensitivity to sampling steps (S_T)

> **[Comment-5]** Abbreviations like AR need to be introduced before first used.

Response: Thank you for the helpful suggestion. We have revised the manuscript to introduce the abbreviation at its first occurrence. Specifically in the Introduction (Line 24 in the revised manuscript). In addition, under the Methods section, the subsection title has been updated to *"Autoregressive (AR) Steps Formulation"* (Line 402 in the revised manuscript) for more clarity.

> **[Comment-6]** It is repeatedly highlighted that Mol-CADiff generates valid molecules, however, this seems be because of the choice of (external) graph decoder and therefore should be less highlighted.

Response: Thank you for this helpful comment. We agree that the chemical validity of generated molecules is largely ensured by the graph decoder, which provides structurally

consistent graph reconstruction. Following your helpful suggestion, we have revised the manuscript so that this point is now less highlighted. The revised parts are:

Previous: This integration of autoregression and diffusion enables the model to capture structural dependencies while effectively generating chemically valid, novel, and diverse molecular graphs (Lines 148-149 in prior submission).

Revised: This integration of autoregression and diffusion enables the model to capture structural dependencies while effectively generating novel and diverse molecular graphs (Lines 389-390 in the revised manuscript).

Previous: This visual similarity underscores our method's ability to effectively leverage textual conditions, resulting in chemically valid molecules that reflect the desired properties (Lines 258-260 in prior submission).

Revised: This visual similarity underscores our method's ability to effectively leverage textual conditions that reflect the desired properties (Lines 155-156 in the revised manuscript).

Conclusion

We appreciate the constructive feedback from the reviewers and believe that the manuscript has been improved as a result. We have addressed all the concerns raised and made the necessary revisions.

References

[1] C. Deng, D. Zh, K. Li, S. Guan, H. Fan, Causal diffusion transformers for generative modeling, arXiv preprint arXiv:2412.12095 (2024).

[2] H. Zhu, T. Xiao, V. G. Honavar, 3m-diffusion: Latent multi-modal diffusion for language-guided molecular structure generation, in: First Conference on Language Modeling, 2024.

[3] Y. Zhang, G. Ye, C. Yuan, B. Han, L.-K. Huang, J. Yao, W. Liu, Y. Rong, Atomas: Hierarchical adaptive alignment on molecule-text for unified molecule understanding and generation, in: The Thirteenth International Conference on Learning Representations, 2025.
URL `https://openreview.net/forum?id=mun3bGqdDM`

[4] J. Li, Y. Liu, W. Fan, X.-Y. Wei, H. Liu, J. Tang, Q. Li, Empowering molecule discovery for molecule-caption translation with large language models: A chatgpt perspective, IEEE transactions on knowledge and data engineering 36 (11) (2024) 6071–6083.

[5] C. Vignac, I. Krawczuk, A. Siraudin, B. Wang, V. Cevher, P. Frossard, Digress: Discrete denoising diffusion for graph generation, in: The Eleventh International Conference on

Learning Representations, 2023.
URL https://openreview.net/forum?id=UaAD-Nu86WX

[6] J. Jo, D. Kim, S. J. Hwang, Graph generation with diffusion mixture, in: Proceedings of the 41st International Conference on Machine Learning, 2024, pp. 22371–22405.
